# 3D Carbon Frameworks for Ultrafast Charge/Discharge Rate Supercapacitors with High Energy-Power Density

**DOI:** 10.1007/s40820-020-00535-w

**Published:** 2020-10-27

**Authors:** Changyu Leng, Zongbin Zhao, Yinzhou Song, Lulu Sun, Zhuangjun Fan, Yongzhen Yang, Xuguang Liu, Xuzhen Wang, Jieshan Qiu

**Affiliations:** 1 State Key Lab of Fine Chemicals, School of Chemical Engineering, Liaoning Key Lab for Energy Materials and Chemical Engineering, Dalian University of Technology, Dalian, 116024 People’s Republic of China; 2 College of Chemical Engineering, Beijing University of Chemical Technology, Beijing, 100029 People’s Republic of China; 3 School of Materials Science and Engineering, China University of Petroleum, Qingdao, 266580 Shandong People’s Republic of China; 4 Key Lab of Interface Science and Engineering in Advanced Materials, Ministry of Education, Taiyuan University of Technology, Taiyuan, 030024 People’s Republic of China

**Keywords:** 3D carbon frameworks, Nanocages, Ultrafast charge/discharge rate, High energy-power density, Supercapacitors

## Abstract

Carbon-based electric double layer capacitors (EDLCs) hold tremendous potentials due to their high-power performance and excellent cycle stability. However, the practical use of EDLCs is limited by the low energy density in aqueous electrolyte and sluggish diffusion kinetics in organic or/and ionic liquids electrolyte. Herein, 3D carbon frameworks (3DCFs) constructed by interconnected nanocages (10–20 nm) with an ultrathin wall of ca. 2 nm have been fabricated, which possess high specific surface area, hierarchical porosity and good conductive network. After deoxidization, the deoxidized 3DCF (3DCF-DO) exhibits a record low IR drop of 0.064 V at 100 A g^−1^ and ultrafast charge/discharge rate up to 10 V s^−1^. The related device can be charged up to 77.4% of its maximum capacitance in 0.65 s at 100 A g^−1^ in 6 M KOH. It has been found that the 3DCF-DO has a great affinity to EMIMBF_4_, resulting in a high specific capacitance of 174 F g^−1^ at 1 A g^−1^, and a high energy density of 34 Wh kg^−1^ at an ultrahigh power density of 150 kW kg^−1^ at 4 V after a fast charge in 1.11 s. This work provides a facile fabrication of novel 3D carbon frameworks for supercapacitors with ultrafast charge/discharge rate and high energy-power density.

**Electronic supplementary material:**

The online version of this article (10.1007/s40820-020-00535-w) contains supplementary material, which is available to authorized users.

## Highlights

- 3D carbon frameworks (3DCFs) constructed by interconnected nanocages show a high specific surface area, hierarchical porosity, and conductive network.
- The deoxidization process removed most of surface oxygen-containing groups in 3DCFs that leads to fast ion diffusion kinetics, good electric conductivity, and limited side reactions.
- The deoxidized 3DCFs exhibit an ultrafast charge/discharge rate as electrodes for SCs with high energy-power density in both aqueous and ionic liquids electrolytes.

**Electronic supplementary material:**

The online version of this article (10.1007/s40820-020-00535-w) contains supplementary material, which is available to authorized users.

## Graphical abstract

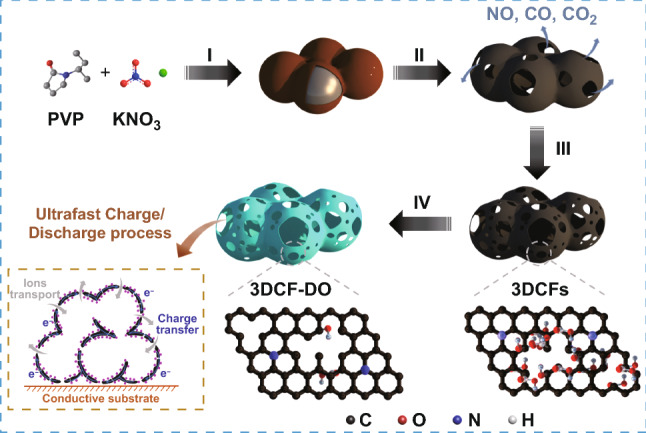

## Introduction

Due to the high power density and long cycle life, carbon-based supercapacitors (SCs) have many applications in energy storage fields, e.g., power grid, portable devices and electric vehicles [1]. Carbon electrode materials are one of the most important factors that govern the performance of SCs. Up to now, considerable efforts have been made aiming at developing advanced carbon materials for SCs with high energy density. 0D Carbon dots [2], 1D carbon nanotubes [3], and 2D graphenes [4] were all explored as electrodes for high performance SCs. In particular, 3D carbon frameworks with high specific surface area, hierarchical porosity and large conductive network are considered as promising electrodes for SCs [5–9]. Carbon nanocages with accessible active sites, thin carbon walls and small hollow spaces can provide high specific capacitance, shorten the transport length for ions and buffer the stress from the expansion and shrinkage during the charge/discharge process [10–14]. However, the isolated carbon nanocages without continuous networks may reduce the electric conductivity as electrodes, leading to slow charge transfer. Therefore, the construction of 3D carbon frameworks with large and continuous conductive network made of carbon nanocages still remains a challenge.

For carbon materials, the surface properties also have a remarkable impact on their capacitive performance [7, 15–21]. It has been demonstrated that surface oxygen functional groups are beneficial to the wettability of carbon electrode surface and provide additional pseudocapacitance in aqueous electrolyte [22]. However, high surface oxygen content will decrease the electric conductivity of carbon electrodes, cause rapid capacity loss at high current density and generate polarization reaction at high voltage during the charge/discharge process, which should be avoided [23]. All in all, the understanding of the relationship between surface properties of carbon electrodes and electrochemical performance is critical for the development of SCs [24–26].

To date, there is an urgent need for developing SCs with high energy density because the low energy density (5–10 Wh kg^−1^) of SCs cannot meet the ever growing demands of energy storage [27–29]. According to the equation *E *=1/2 CV^*2*^, the energy density (*E*) of SCs can be improved by increasing the specific capacitance (*C*) and/or operation voltage (*V*) [30]. Major strategies to improve *C* are realized by exploring redox-active materials (such as conducting polymers [31–33], transition metal oxides [34–36], hydroxides [37–39]) to contribute additional pseudocapacitance or designing hybrid supercapacitors (HSCs) [40–42] and asymmetric supercapacitors (ASCs) [42–44]. But these strategies cause the sabotaging of the rate performance and cycling stability of SCs inevitably. On the other hand, enlarging *V* is considered as a more effective tactic for improving the energy density of carbon-based SCs. The voltage window of SCs can be easily widened by using organic and ion liquids (ILs) electrolytes (3–4.4 V) [45–49]. However, ILs are seriously plagued by their intrinsic large ion size, high viscosity and sluggish diffusion kinetics that will decrease the power density of SCs. The suitable pore size distribution, ideal surface properties and excellent electric conductivity of carbon electrode materials can improve the ion transport and electron transfer to ameliorate sluggish diffusion kinetics of ILs, which can provide a high energy density without sacrificing the rapid energy storage rate for SCs [50].

Herein, we have proposed a simple strategy involving one-step gas foaming and in situ activation to fabricate 3D carbon framework constructed by continuous nanocages with high specific surface area, hierarchical porosity and large conductive networks. After deoxidization, the deoxidized 3DCF showed an ultrafast charge/discharge rate in both aqueous and ILs electrolytes and high energy density while maintaining high power density in ILs electrolyte, suggesting a promising electrode for high-performance SCs.

## Experimental

### Synthesis of 3DCF Materials

Polyvinylpyrrolidone (10 g, PVP k30) was dispersed into deionized water at room temperature, followed by adding 7.5 g KNO_3_ under stirring to form clear solution without any suspended solid. After that, the solution was transferred into a container and placed into a refrigerator at − 20 °C for 24 h until fully frozen. Then the ice cake was put in a lyophilizer at − 80 °C at 1.0 Pa for 3 days, yielding dry white powders. Next, the precursor was put into a corundum boat and heated at a rate of 10 °C min^−1^ in pure Ar with a flow rate of 200 mL min^−1^ and kept at different temperatures (700, 800, and 900 °C) for 1 h, yielding black products that were then washed by deionized water and 1 M HCl solution until pH = 7. Finally, the materials were dried in oven at 120 °C for 12 h, named as 3DCF-X (X refers to the final temperature of the annealing process). The deoxidized 3DCFs (3DCF-DO) were obtained by further annealing 3DCF-900 at 900 °C for 1 h in mixed H_2_/Ar atmosphere (5% H_2_, in volume ratio) to eliminate most surface oxygen functional groups.

### Characterization

Thermogravimetric analysis (TG) was performed on a thermal analyzer (TA-Q50) to explore temperature of chemical blowing and in situ activation process. The surface morphology and internal structure of samples were examined by the scanning electron microscopy (FESEM) and transmission electron microscopy (TEM), respectively. X-ray diffraction (XRD) and Raman analysis were carried out to analyze the crystallinity and graphitic nature of the materials. The porous texture of the obtained materials was analyzed by a nitrogen adsorption/desorption technique. XPS analysis was carried out to analyze the elements of the materials. Please refer to the Supplementary File for details.

### Electrochemical Test of 3DCF Electrodes

All electrodes were prepared by a mixture of 5 wt.% of PVDF, 10 wt.% of acetylene black and 85 wt.% of as-obtained materials. The mixture was pressed on the Ni foam at 10.0 MPa. The two-electrode symmetric devices were fabricated in 6 M KOH and EMIMBF_4_, respectively. All ILs-based two-electrode cells were assembled in an Ar-filled dry glove-box (MIKROUNA, with < 1 ppm of O_2_ and H_2_O). Two electrodes slices with similar mass loadings (~ 2, 5, and 10 mg) were directly placed inside the CR2026 coin-type cells and separated. Galvanostatic charge/discharge curve (GCD), cyclic voltammetry (CV) and electrochemical impedance spectroscopy (EIS) were carried out on the electrochemical workstation (Bio-Logic, VP3, France).

## Results and Discussion

### Structure Characterization

**Fig. 1:**
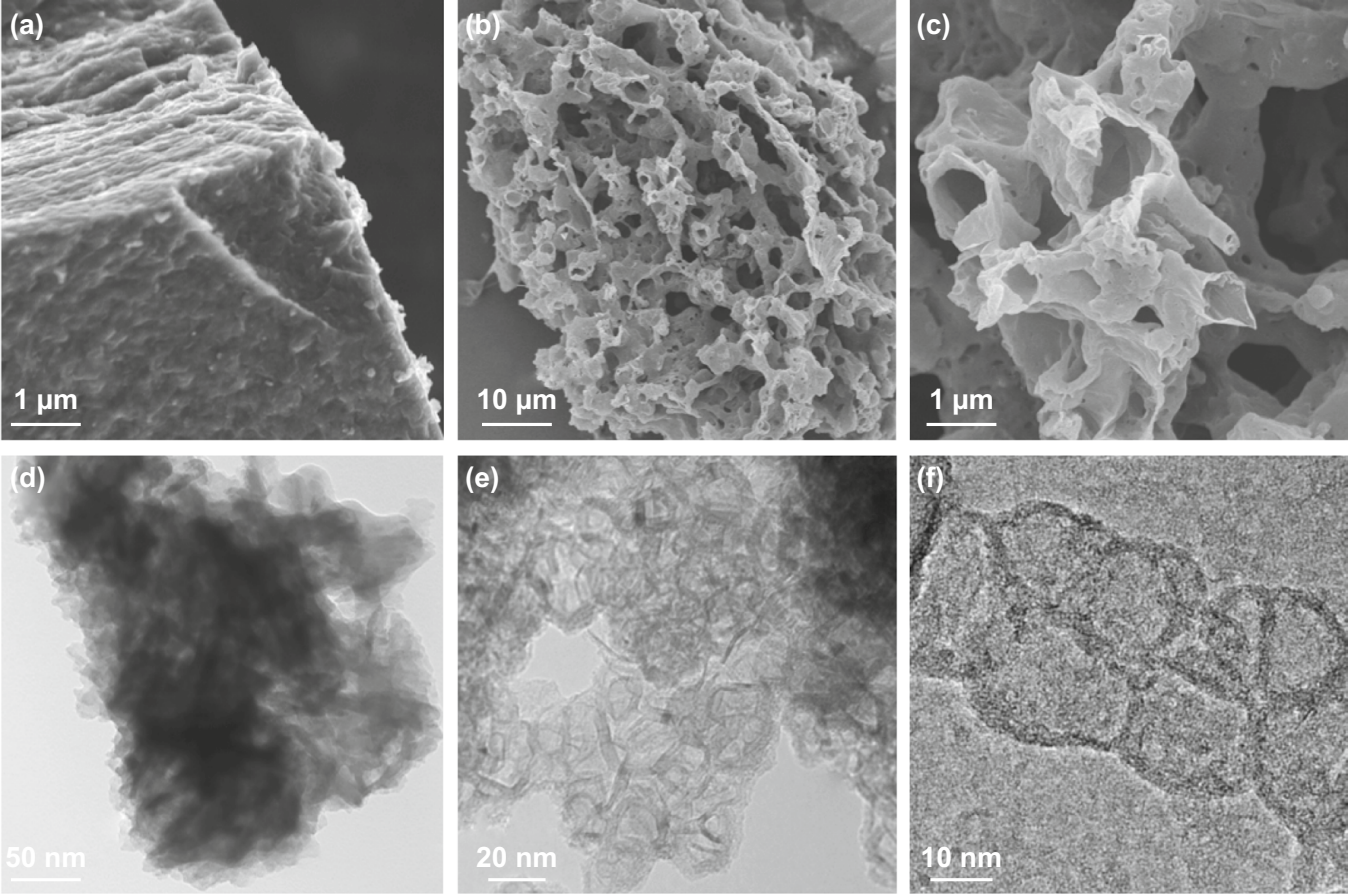
SEM images of **a** pure PVP-derived carbon and **b**, **c** 3DCFs. TEM images of **d** pure PVP-derived carbon and **e** 3DCFs. **f** High-resolution TEM image of 3DCFs consisting of continuous nanocages (10–20 nm) with ultrathin walls (ca. 2 nm)

The surface morphology and nanostructures of 3DCF materials were characterized by scanning electron microscopy (SEM) and transmission electron microscopy (TEM) (Fig. 1). It can be seen that the direct carbonization of pure PVP has yielded a bulk and compact materials (Fig. 1a), while the pyrolysis of PVP/KNO_3_ resulted in porous and loose materials (Fig. 1b, c). As shown by the TEM images (Fig. 1d, e), the 3DCF materials are made of continuous nanocages (10-20 nm) with ultrathin walls (ca. 2 nm). The HR-TEM observation has clearly revealed that the interconnected nanocages have micro-/mesopores on their walls (Fig. 1f). The morphologies and formation mechanism of 3DCFs fabricated by traditional KOH activation and in situ activation are shown and compared (Figs. S6, S7). The traditional KOH activation destroys the continuous frameworks irreversibly, while the present in situ activation (inside-out activation) creates homogeneous micropores on the walls of adjacent nanocages. In other words, KNO_3_ results in both gas foaming and in situ activation, forming continuous nanocages and a large number of micro-/mesopores.

**Scheme 1:**
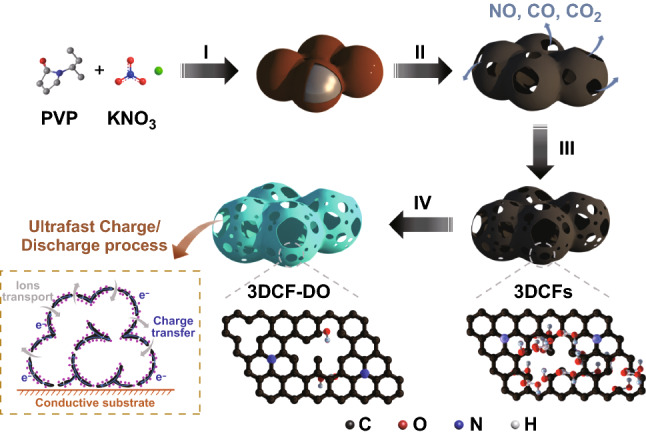
Schematic illustration of the synthesis of 3DCF materials via (I) thermal melting, (II) gas foaming, (III) in situ activation and (IV) deoxidization processes

The formation of 3DCFs with continuous nanocages includes a series of processes: thermal melting, pyrogenic decomposition, in situ activation and deoxidization. The synthetic protocol (Scheme 1) is strongly evidenced by TG-MS analysis (Fig. S1). It can be seen from Fig. S1a that the pyrolysis temperature of PVP, KNO_3_, and PVP/KNO_3_ is 450, 600, and 375 °C, respectively. The lower pyrolysis temperature (375 °C) of PVP/KNO_3_ compared with pure PVP demonstrates that KNO_3_ effectively promotes the pyrolysis of PVP, indicating the strong interaction between PVP and KNO_3_. According to the TG-MS analysis, the rapid mass loss and a large amount of gases release (NO, CO, CO_2_) have simultaneously taken place at about 375 °C, ascribed to the gas foaming process. Subsequently, the foamed polymer is converted to carbon materials at the elevated pyrolysis temperature, indicated by the mass loss peak at about 450 °C (Fig. S1b). It should be noted that another mass loss peak (Fig. S1a) and CO emission peak (Fig. S1c) appeared at about 700 °C, which can be attributed to the activation of carbon by K^+^ derived from KNO_3_. As a result, abundant micro-/mesopores were created during the in situ activation process. The universality of this strategy was confirmed by using other alkali metal nitrates, such as NaNO_3_ and LiNO_3_ (Figs. S2, S3), 3DCF materials were also obtained with these alkali metal nitrates (Figs. S4, S5). Finally, the oxygen functional groups on the surface of as-obtained 3DCFs were removed in H_2_/Ar mixed atmosphere (5% H_2_) at 900 °C.

**Fig. 2:**
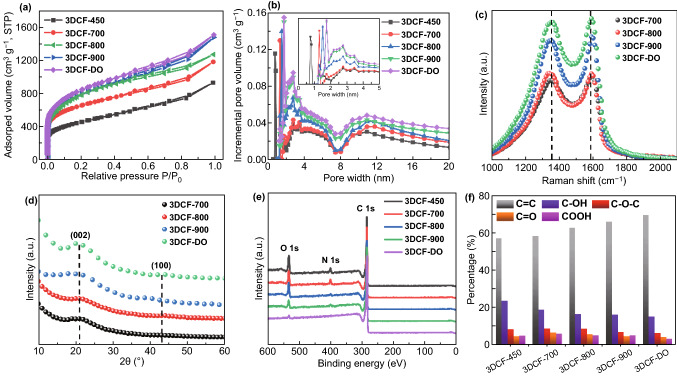
**a** N_2_ adsorption and desorption isotherms, **b** corresponding pore size distributions, **c** Raman spectra, **d** powder XRD patterns, **e** survey spectra and **f** C 1*s* deconvoluted spectra of 3DCF materials

The porous structure of the obtained 3DCF materials was studied by N_2_ adsorption–desorption isotherm measurements. As shown in Fig. 2a, all these materials showed a type IV adsorption–desorption isotherm with a hysteresis loop at the relative pressure from 0.4 to 1.0 in the desorption branch, indicating multiple scale pores of 3DCFs. The 3DCF-450 exhibited the Brunauer–Emmett–Teller (BET) surface area of 1502 m^2^ g^−1^ mainly resulting from the foaming process. With the rise in temperature, the SSA of 3DCF-700, 3DCF-800, and 3DCF-900 increases from 2098 to 2592, 2602 m^2^ g^−1^, respectively. The higher SSA is due to the activated etching of the carbon frameworks by K^+^ formed from the pyrolysis of KNO_3_. In addition, the *V*_meso_/*V*_micro_ ratio of 3DCF-700, 3DCF-800 and 3DCF-900 increases gradually from 1.2 to 1.4 and 2.2 (Table S1), respectively, indicating that micropores (0.5–1 nm) in 3DCFs were extended to mesopores with increasing temperature. The pore size distribution (PSD) of 3DCF materials is mainly centered at 1–2, 2–5, and 8–20 nm (Fig. 2b). The numerous micropores are formed by the in situ activation of K^+^, and mesopores are further expanded from micropores due to the etching effect of CO_2_. The mesopores of 3DCF-DO can provide accessible space for rapid ion buffer and energy storage; this is especially the case with EMI^+^ ions (~ 0.7 nm) as electrolyte. Overall, the hierarchical porosity of 3DCFs is not only beneficial to energy storage and rapid ion transfer, but also shortening path of ion diffusion [47]. After deoxidization of 3DCF-900 at 900 °C in H_2_/Ar mixed atmosphere, the as-formed 3DCF-DO is very similar to 3DCF-900 in terms of SSA and micropores, suggesting the robust structure at high temperature. The crystallinity and graphitic nature of 3DCFs are examined by X-ray diffraction and Raman analysis, of which the results are shown in Fig. 2c, d. The XRD patterns of the materials show two typical diffraction peaks at 2*θ* = 22°–24° and 42°–44°, corresponding to (002) and (100) planes of graphite, respectively. All patterns exhibit broad (002) diffraction peak, suggesting the amorphous structure of 3DCF materials. The (002) peak of 3DCF-DO is higher than other 3DCFs, indicating that the crystallization is enhanced during the deoxidization process. According to the Bragg equation [51], the interlayer spacing of 3DCF-DO is calculated to be about 0.43 nm for (002) plane, which is larger than 0.335 nm of the typical graphite layer [52]. Raman analysis of 3DCF materials shows two typical Raman peaks at 1350 cm^−1^ (D band) and 1580 cm^−1^ (G band). The *I*_G_/*I*_D_ ratio of 3DCF-DO (1.08) is the highest among these materials, which is consistent with the XRD results mentioned above.

The content and chemical state of C, N, and O in 3DCF materials were analyzed by XPS, as shown in Fig. 2e. For 3DCFs prepared at different annealing temperature of 450, 700, 800, and 900 °C, the oxygen content is 11.33, 8.63, 7.90, and 5.80 at.%, respectively (Table S2). After the deoxidization process, the surface oxygen content of 3DCF-DO further dropped to 1.69 at.%, indicating that most of oxygen on the surface of 3DCF-900 has been eliminated effectively by annealing at high temperature in H_2_/Ar mixed atmosphere. The XPS survey spectrum of 3DCFs in Fig. 2e reveals the peaks of C 1*s*, O 1*s*, and N 1*s*, indicative of the self-doping of O, N in the materials. The C 1*s* spectrum of 3DCF materials is deconvolved into five peaks at 284.5, 285.4, 286.8, 287.3, and 288.8 eV (Fig. S9), corresponding to C=C, C–OH, C–O–C, C=O, and O=C–OH, respectively. As shown in Fig. 2f, the C–OH content sharply decreases from 23.8 to 15.4% with temperature rise. Meanwhile, other O species, such as C–O–C (8.6–6.5%), O=C–OH (5.2–3.5%) and C=O (4.9–4.5%), are also reduced. It can be attributed to the easier removal of O=C–OH and C–OH distributed in edges of materials than those C–O–C and C=O within carbon planes [7] (Table S3). Generally, the O doping of carbon electrodes is regarded as desirable in aqueous electrolytes for additional reversible pseudocapacitance. However, excess oxygen in the carbon electrode can cause serious problems such as gas evolution (CO, CO_2_) and polarization during the charge/discharge process in organic or ILs electrolyte [7]. Therefore, the elimination of surface O functional groups is necessary for carbon materials working under high working potential windows (> 3 V) [23]. N doping is known to affect the electrochemical performance of carbon materials [52]. In our case, after deoxidization, the content of N in 3DCFs is slightly reduced from 1.11 to 0.86%, which is assumed to have slight impact on the electrochemical performance of 3DCF electrodes (Fig. S10).

### Electrochemical Performance

**Fig. 3:**
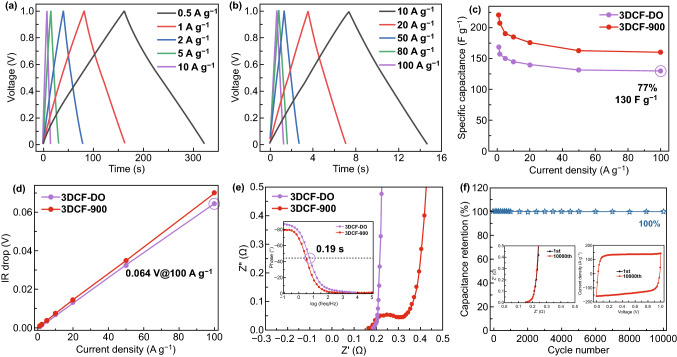
Electrochemical performance of 3DCF-900 and 3DCF-DO (before and after deoxidization) as electrodes of symmetric two-electrode coin cells in 6 M KOH. **a** GCD curves of 3DCF-DO at current density from 0.5 to 10 A g^−1^. **b** GCD curves at current density from 10 to 100 A g^−1^. **c** Rate performance, **d** IR drop, **e** Nyquist plots (inset is Bode plots) of 3DCF-900 and 3DCF-DO, respectively. **f** Stability test conducted at 1 V s^−1^ for 10,000 cycles (inset is CV curves and Nyquist plots of 1st and 10,000th cycle)

The electrochemical performance of 3DCFs//3DCFs symmetric supercapacitors was tested in 6 M KOH. All GCD curves showed highly symmetric triangle profiles (Fig. 3a). The specific capacitance of 3DCF-DO is 168 F g^−1^, with 78% capacitance retention at a high current density of 100 A g^−1^, in comparison with 70% for 3DCF-900. As shown in Fig. 3d, the voltage drop (IR drop) obtained from the GCD curves of the 3DCF-DO varies linearly with the current density. At the current density of 100 A g^−1^, the IR drop of the 3DCF-DO is as low as 0.064 V, much lower than the values reported previously. Furthermore, the structure-performance relationship of carbon materials is explored by electrochemical impedance spectroscopy (EIS) (Fig. 3e). Compared with 3DCF-900, the Nyquist plot of 3DCF-DO displays a smaller semicircle in the high-frequency region and almost vertical line in the low-frequency region, suggesting smaller *R*_s_, *R*_ct_ and more ideal capacitive behavior.

The Bode phase diagram of 3DCF-DO exhibits a characteristic frequency (*f*_0_) at the phase angle of − 45° of 5.4 Hz, corresponding to a characteristic time constant (*τ*_0_ = 1/*f*_0_) of 0.19 s (inset in Fig. 3e). The rapid frequency response is consistent with the high rate performance of 3DCF-DO. Furthermore, the 3DCF-DO shows an ultrafast charge time of 0.65 s at 100 A g^−1^ and a capacitance retention of 77.4% at the current density of 1 A g^−1^. 3DCF-DO//3DCF-DO symmetric SCs show a satisfactory cycle stability, retaining 100% initial specific capacitance after 10,000 cycles at 1 V s^−1^ in 6 M KOH (Fig. 3f). The CV of 3DCF-DO was measured in 6 M KOH ranging from 10 mV s^−1^ to 10 V s^−1^, and the typical EDLC-type curves with ideal rectangular shape were obtained without distortion even at an ultrafast scan rate of 10 V s^−1^ (Fig. S11). It can be seen that the 3DCF-DO shows a specific capacitance of 240, 138, and 132 F g^−1^ at 10 mV s^−1^, 5 V s^−1^, and 10 V s^−1^, respectively. The good linear dependence of the discharge current on the scan rate up to 10 V s^−1^ (*R*^2^ = 0.9996) demonstrates an ultrahigh rate performance for the 3DCF-DO electrode. The CV curves of the 3DCF-DO at the scan rate of 1, 2, and 5 V s^−1^ show almost rectangular shapes (Fig. S11d-f). The above results have suggested the ultrafast charge/discharge rate and high power capability of the 3DCF-DO as electrode for aqueous SCs. The electric conductivity of the materials can affect the charge transfer to some extent. In this regard, the slope of 3DCF-DO is bigger than that of the other samples based on the linear sweep voltammetry (LSV) curves (Fig. S13a), implying its highest electric conductivity from the large conductive networks. The 3DCF-DO electrode keeps a great rate performance even at the mass loading of 10 mg cm^−2^ with a specific capacitance of 142 F g^−1^ at the current density 1 A g^−1^ (85% capacitance retention with the mass loading of 2 mg cm^−2^, Fig. S13b). The results further prove that the thick electrodes of 3DCF-DO have high specific capacitance, outstanding rate performance, and robust electrochemical kinetics, which is required for practical applications.

**Fig. 4:**
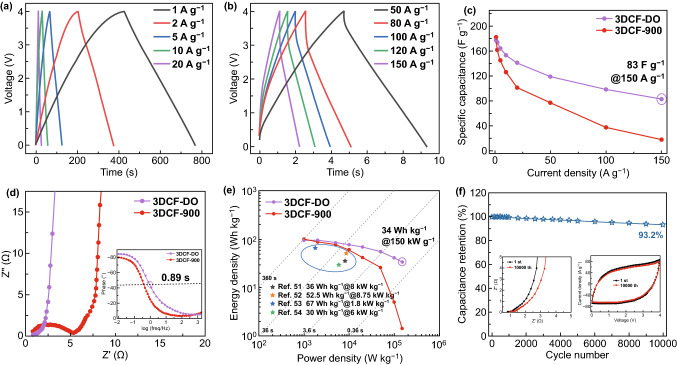
Electrochemical performance of 3DCF-900 and 3DCF-DO as electrodes of symmetric two-electrode coin cells in EMIMBF4 electrolyte at 4 V; **a** GCD curves of 3DCF-DO at current density from 1 to 20 A g^−1^; **b** GCD curves of 3DCF-DO at current density from 50 to 150 A g^−1^; **c** Rate performance, **d** Nyquist plots (inset is the Bode plots). **e** Ragone plots of 3DCF-900 and 3DCF-DO. **f** Stability test conducted at 1 V s^−1^ for 10,000 cycles in EMIMBF4 at 4 V

3DCFs//3DCFs symmetric two-electrode cells using EMIMBF_4_ as electrolyte were tested at a high voltage of 4 V, as shown in Fig. 4. The relationship between surface deoxidization and electrochemical performance of 3DCF electrodes is more significant in EMIMBF4 ILs. The capacitive performance of 3DCF-DO is demonstrated from the GCD curves in Fig. 4a, b. Accordingly, the calculated specific capacitance of 3DCF-DO is 174, 141, 98, and 83 F g^−1^ at the current densities of 1, 10, 100, and 150 A g^−1^, respectively (Fig. 4c). An ultrafast discharge time of 1.96 s at 100 A g^−1^ and a capacitance retention of 56% at the current density of 1 A g^−1^ indicate the superb rate capability of 3DCF-DO, while the capacity retention of 3DCF-900 is only 20%. The capacitive performance of 3DCF-700, 3DCF-800, and 3DCF-900 as electrodes is investigated and compared in ILs electrolyte (Fig. S14). The electrochemical properties of these 3DCFs are improved with the temperature rise. Compared with 3DCFs without deoxidization, the 3DCF-DO has shown the highest rate performance and the smallest IR drop at 100 A g^−1^ (Fig. S15). In addition, 3DCF-DO as electrode for SC is tested in EMIMBF_4_ at different potential windows, the charge curves of GCD at 3, 3.5, and 4 V overlap each other fully (Fig. S16). These excellent electrochemical properties can be attributed to the low oxygen content of 3DCF-DO, which limits gas evolution and polarization to ensure the high rate capability and cycle stability at 4.0 V. The 3DCF-DO exhibits the smaller *R*_s_, *R*_ct_, and ESR than the 3DCF-900 in Fig. 4d. The Nyquist plots of 3DCF-DO reveal a vertical low-frequency line, a high-frequency semicircle region and 45°-slop mid-frequency Warburg region with the knee *f*_0_ of 1.12 Hz corresponding to a characteristic time constant *τ*_0_ of 0.89 s. The charge transfer resistance (*R*_ct_) and total internal resistance (*R*_s_) of 3DCF-DO are 0.18 and 0.80 Ω, respectively, which proves the rapid diffusion kinetics and high electric conductivity of the 3DCF-DO electrode in EMIMBF_4_ ILs electrolyte (Tables S4, S5). In Fig. 4e, the energy densities of 3DCF-900 and 3DCF-DO are acquired in Ragone plots in the range of power density from 1 to 150 kW kg^−1^. The 3DCF-900 shows an energy density of 100 Wh kg^−1^ at 1 kW kg^−1^ but only preserved 1.46 Wh kg^−1^ at 150 kW kg^−1^. However, the 3DCF-DO shows 97 Wh kg^−1^ at 1 kW kg^−1^ and still remains a high energy-power density of 34 Wh kg^−1^ at 150 kW kg^−1^ (~ boosted 23-fold in performance after deoxidization) for 4 V EMIMBF_4_-based SC. A capacitance retention of 93.2%, only 0.068% decay per cycle, at a scan rate of 1 V s^−1^ for 10,000 cycles is obtained for 3DCF-DO//3DCF-DO symmetric SCs in EMIMBF_4_ at 4 V (Fig. 4f). These results have indicated that the 3DCF-DO is a promising electrode to deliver ultrafast charge/discharge rate and high energy density at ultrahigh power density in EMIMBF_4_ electrolyte. The Ragone plots of 3DCF-DO//3DCF-DO symmetric SCs are compared with reported symmetric SCs in the literature. Obviously, the electrochemical properties of 3DCF-DO have exceeded most of the carbon-based electrodes for SCs in aqueous and ILs electrolytes (Tables S6, S7).

The excellent electrochemical properties of 3DCF-DO are not only attributed to the 3D carbon framework with continuous nanocages to provide large conductive networks for rapid charge transfer, but also the elimination of surface oxygen groups, which results in the following benefits: (a) the removal of surface O groups on the pore openings makes the easy entrance of electrolyte ions and thus improve the ion diffusion kinetics during the charge/discharge process; (b) the reduction of surface O groups improves the electric conductivity of materials and boosts the rapid electron transfer; (c) the removal of surface O groups limits gas evolution and various side reactions to keep material’s stability as electrode especially working at 4.0 V high potential windows.

## Conclusions

In summary, the 3D carbon frameworks with continuous nanocages have been fabricated via a combined process of gas foaming, in situ activation and deoxidization. The deoxidized 3DCF shows high specific surface area, continuous conductive network with multiple scale pores, and ultralow surface O content. As electrode for aqueous SCs, the deoxidized 3DCFs show an ultrafast charge/discharge rate, which can be charged up to 77.4% of its maximum capacitance in 0.65 s at 100 A g^−1^. The deoxidized 3DCFs can deliver a high energy density of 34 Wh kg^−1^ at an ultrahigh power density of 150 kW kg^−1^ in 1.11 s in EMIMBF_4_ electrolyte at 4 V. Our strategy provides a pathway to the preparation of novel 3D carbon frameworks for ultrafast charge/discharge rate SCs with high energy-power density.

## Electronic supplementary material

Below is the link to the electronic supplementary material.

Supplementary material 1 (DOC 5926 kb)
