# Supplementary material for: 3D Carbon Frameworks for Ultrafast Charge/Discharge Rate Supercapacitors with High Energy-Power Density
Source: Nanomicro Lett. 2020 Oct 27;13:8. doi: 10.1007/s40820-020-00535-w (PMC8187691; doi:10.1007/s40820-020-00535-w)
Supplement: Supplementary file 1 — Supplementary material 1 (DOC 5926 kb) [file 40820_2020_535_MOESM1_ESM.doc]

Supporting information

**3D Carbon Frameworks for Ultrafast Charge/Discharge Rate Supercapacitors with High Energy-Power Density**

Changyu Leng^1^, Zongbin Zhao^1^,*, Yinzhou Song^1^, Lulu Sun^1^, Zhuangjun Fan^3^, Yongzhen Yang^4^, Xuguang Liu^4^, Xuzhen Wang^1^ and Jieshan Qiu^1,2^,*

^1^State Key Lab of Fine Chemicals, School of Chemical Engineering, Liaoning Key Lab for Energy Materials and Chemical Engineering, Dalian University of Technology, Dalian 116024, China

^2^College of Chemical Engineering, Beijing University of Chemical Technology, Beijing 100029, China

^3^School of Materials Science and Engineering, China University of Petroleum, Qingdao, 266580, Shandong, PR China

^4^Key Lab of Interface Science and Engineering in Advanced Materials, Ministry of Education, Taiyuan University of Technology, Taiyuan 030024, China

*Corresponding author. E-mail: [zbzhao@dlut.edu.cn](mailto:zbzhao@dlut.edu.cn), [jqiu@dlut.edu.cn](mailto:jqiu@dlut.edu.cn).

**This PDF file includes:**

Experimental Section

Fig. S1 to S16

Tables S1 and S8

References and notes

**Table of Contents**

1. **Experimental Section**
2. **Fig. S1**: TG, DTG and TG-MS analysis of PVP, KNO_3_ and PVP/KNO_3_.
3. **Fig. S2**: TG, DTG and TG-MS analysis of PVP, NaNO_3_ and PVP/NaNO_3_.
4. **Fig. S3**: TG, DTG and TG-MS analysis of PVP, LiNO_3_ and PVP/LiNO_3_.
5. **Fig. S4**: SEM images of 3DCF-900, 3DCF-Na-900 and 3DCF-Li-900.
6. **Fig. S5**: TEM images of 3DCF-900, 3DCF-Na-900 and 3DCF-Li-900.
7. **Fig. S6**: SEM images of 3DCF prepared by *in-situ* activation and KOH activation.
8. **Fig. S7**: The structural difference between mechanism of *in-situ* activation and traditional outside-in KOH activation.
9. **Fig. S8**: The proposed ion diffusion models of EMI**^+^** in pores with different size.
10. **Fig. S9**: C 1s deconvoluted spectra of 3DCF materials.
11. **Fig. S10**: N 1s deconvoluted spectra of 3DCF materials.
12. **Fig. S11.** Cyclic voltammetry performance of 3DCF-DO as electrode of symmetric two-electrode coin cell in 6 _M_ KOH.
13. **Fig. S12**: Electrochemical performance of 3DCF-Li and 3DCF-Na materials measured in a two-electrode system in 6 M KOH electrolyte.
14. **Fig. S13.** The relationship between interconnected nanocages and corresponding materials properties.
15. **Fig. S14**: Rate performance of 3DCF materials in 6 M KOH and EMIMBF_4_ electrolytes.
16. **Fig. S15**: Electrochemical performance of 3DCF materials in the symmetric two-electrode coin cells in EMIMBF_4_ at 4 V
17. **Fig. S16**: 3DCF-DO-based supercapacitor at different potential windows of 3 V, 3.5 V, 4 V in EMIMBF_4_ electrolyte.
18. **Table S1**: The content of C, N, O in 3DCF materials according to the XPS.
19. **Table S2**: C1s analysis of 3DCF materials.
20. **Table S3**: Structural characteristics of 3DCF materials.
21. **Table S4**: Various resistances of the 3DCF materials in ILs electrolyte.
22. **Table S5**: The corresponding relaxation time constant (τ) of the 3DCF materials.
23. **Table S6**: Comparison between 3DCFs and other advanced carbon electrodes for aqueous SCs.
24. **Table S7**: Comparison between 3DCFs and other advanced carbon electrodes for ILs-based SCs.
25. **Table S8**: Comparison of mass loading performance for various carbon-based SCs in aqueous electrolytes.
26. **References and notes**

**Experimental Section**

**Materials synthesis**

Synthesis of 3DCF-Na and 3DCF-Li: For comparison, 3DCF-Na and 3DCF-Li materials were also prepared with NaNO_3_ or LiNO_3_ instead of KNO_3_ with the same procedure. These materials were named as 3DCF-Na-Y, 3DCF-Li-Y (Y refer to the final temperature of annealing process).

Synthesis of 3DCF-KOH materials: KNO_3_ was replaced by KOH to prepare activated carbon materials for the comparison of the traditional activation with *in-situ* activation proposed in this paper (Supporting information, Fig. S6-S7).

**Material characterization**

[Thermogravimetric](../../../../../DuoNuo/AppData/Local/Yodao/DeskDict/frame/20160130124644/javascript:void(0)%3B) [Analysis](../../../../../DuoNuo/AppData/Local/Yodao/DeskDict/frame/20160130124644/javascript:void(0)%3B) (TG) was performed on a thermal analyzer (TA-Q50) to explore temperature of chemical blowing and *in-suit* activation process. The surface morphology and internal structure of samples were examined by the scanning electron microscopy (FE-SEM, HITACHI UHR FE-SEM SU8220) and transmission electron microscopy (TEM, HR-TEM FEI Tecnai G2 F30), respectively. X-ray diffraction (XRD) was carried out on the Rigaku D/Max 2400 diffractometer with Cu Kα radiation (λ=1.5406 Å). The porous texture of as-obtained materials was analyzed by Micrometrics ASAP 2020 Surface Area and Porosity Analyzer at 77 K. The specific surface area (SSA) was calculated from the N_2_ isotherm with p/p_0_ in the range 0.1-0.25 by applying BET method. The pore size distribution was calculated by the DFT equation. XPS analysis was carried out on Thermo ESCALAB 250 to analyze the content of C, O, N of obtained materials.

**Electrochemical measurements**

Galvanostatic charge/discharge (GCD), cyclic voltammetry (CV) and electrochemical impedance spectroscopy (EIS) were carried out on the electrochemical workstation (Bio-Logic, VP3, France). The CV scan rate was in a range of 10 mV s^-1^ to 10 V s^-1^ in aqueous electrolyte. The voltage window for ILs electrolyte was set as 4 V. The GCD curves were measured under the current density ranging from 0.5 to 200 A g^-1^ and EIS was performed at frequencies from 100 kHz to 10 mHz. The cycle stability was measured by 10000 cycles. The energy and power densities (Ragone plots) were evaluated from the galvanostatic discharge curves by taking account the total mass of electrode materials. The gravimetric capacitance of samples in the three-electrode system was calculated from discharge curves, according to the equation:^[2-4]^

$$C=I\Delta\text{t}/mV$$

The gravimetric capacitance of samples in the two-electrode cell was calculated from discharge curves, according to the equation:

$$C=2I\Delta\text{t}/m\Delta V$$

where *∆t*, *∆V*, *I* and *m* are the discharging time (s), voltage window after removing ohmic drop (V), current (mA) and the total mass of single carbon electrode (mg).

The energy density was calculated according to the equation:

$$E=C(V-IR)^{2}\text{/28.8}$$

where *C* is the specific capacitance of samples in the two-electrode cell, *V* is the set voltage window (V), *IR* is the IR drop of discharge curves (V).

The power density was estimated using the equation:

$$P=E/\Delta\text{t}$$

Where *E* (Wh kg-1)is the energy density of two-electrode cell, *∆t* is the current (mA).

The volumetric energy density (*Ev*) was estimated using the equation:

$$E\text{v}=E*\rho$$

The volumetric power density (*Pv*) was estimated using the equation:

$$P\text{v}=P*\rho$$

Where *ρ* was the packing density of electrode materials.

The EIS spectra is fitted by the model R(C(RW)) as below:


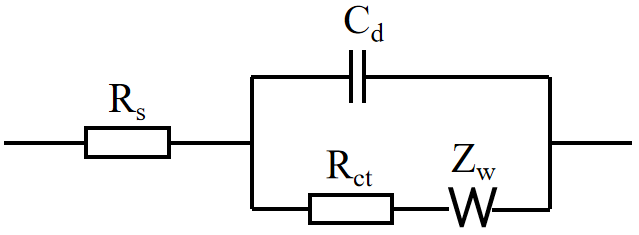


where Rs, Cd, Rct, and Zw represent the solution resistance, double layer capacitance,

charge transfer resistance, and Warburg impedance, respectively.

The equivalent series resistance (ESR) which was obtained according to the equation:

$$\text{ESR}=\text{V}_{\text{drop}}/I$$

where *V_drop_* is the IR drop (V) in the discharge curve.


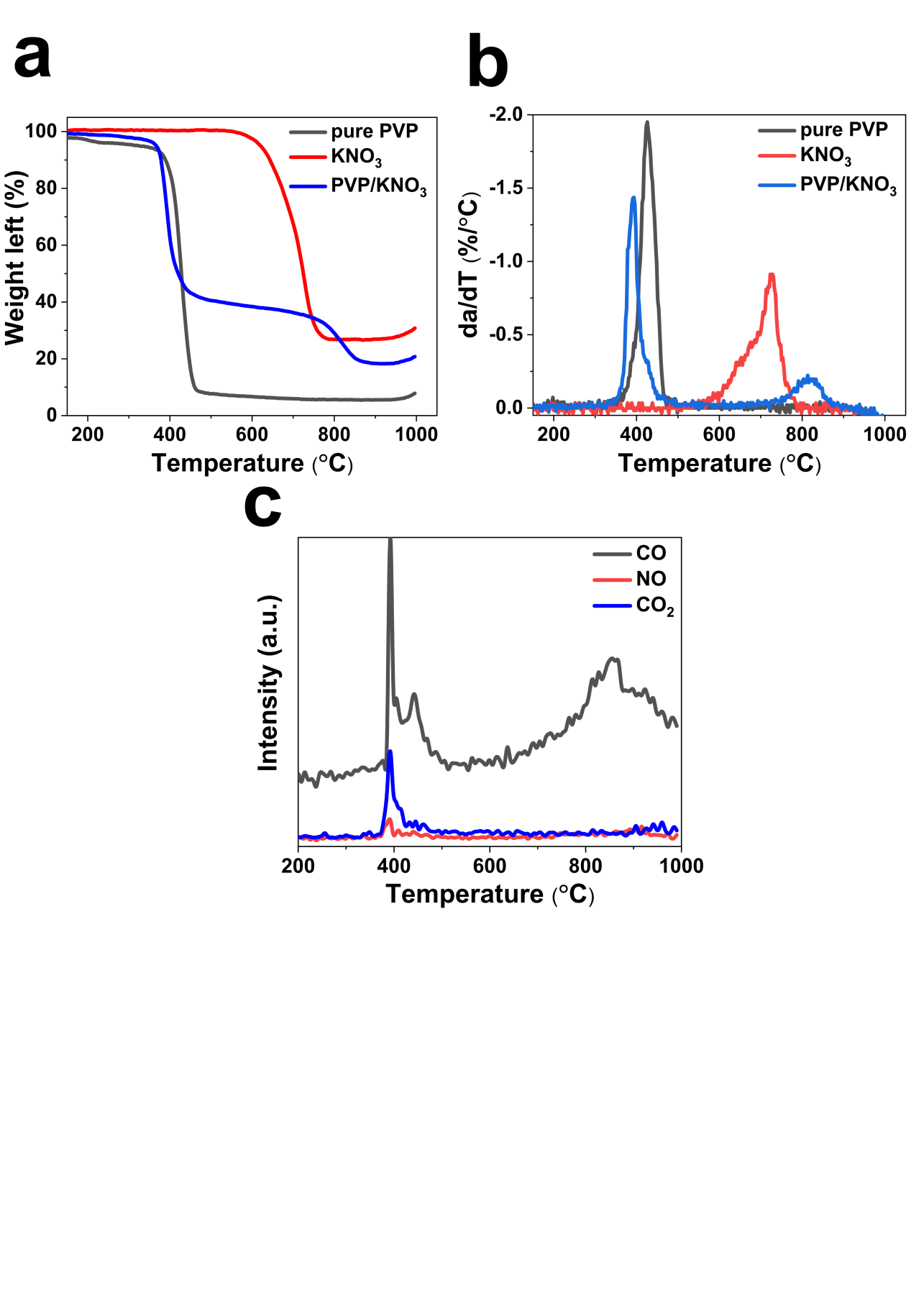


**Fig. S1.** (**a**) TGA and (**b**) DTG curves of PVP, KNO_3_ and PVP/KNO_3_. (**c**) TG-MS of the released gases from the pyrolysis of PVP/KNO_3_.


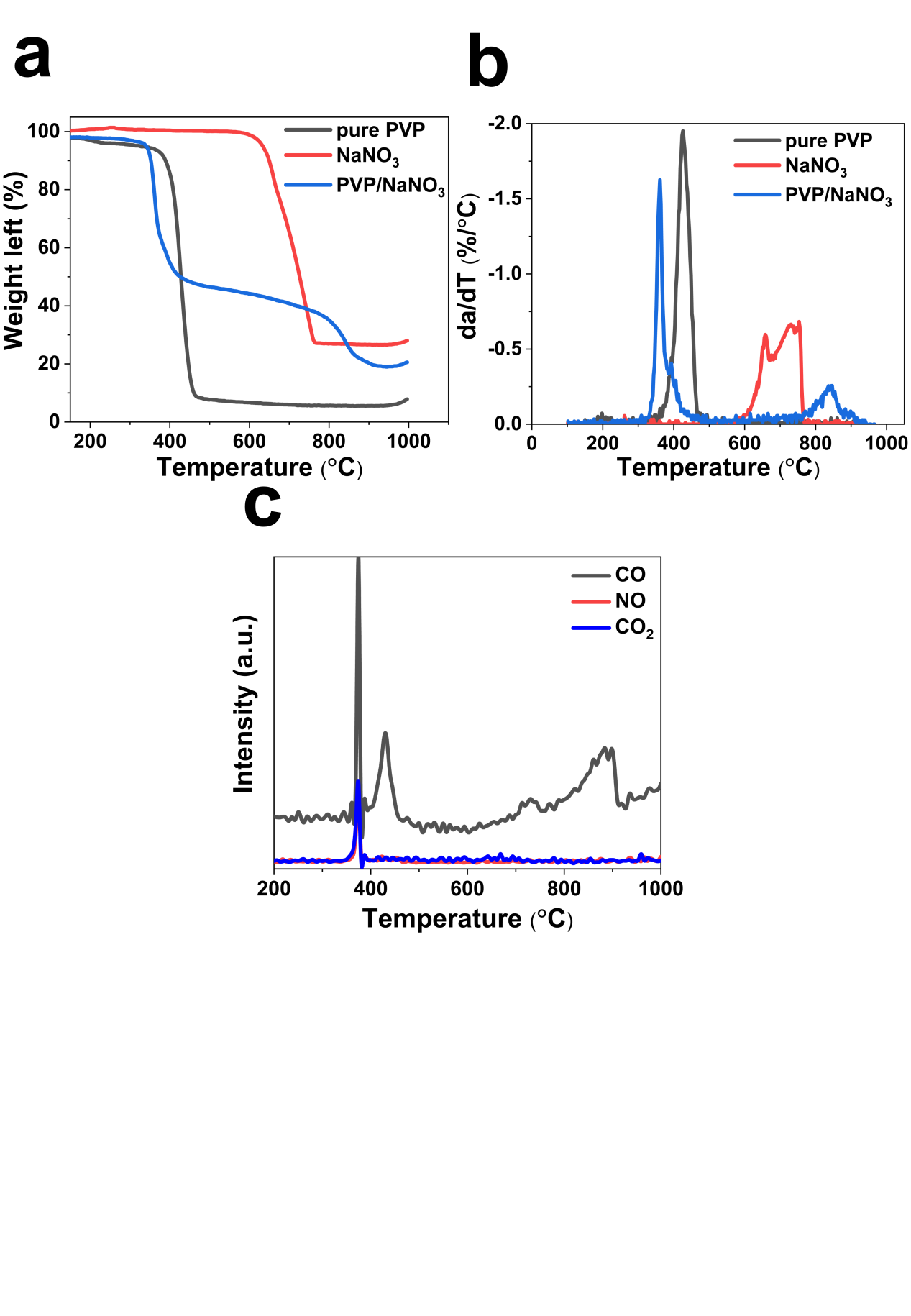


**Fig. S2.** (**a**) TGA and (**b**) DTG curves of PVP, NaNO_3_ and PVP/NaNO_3_. (**c**) TG-MS of the released gases from the pyrolysis of PVP/NaNO_3_.


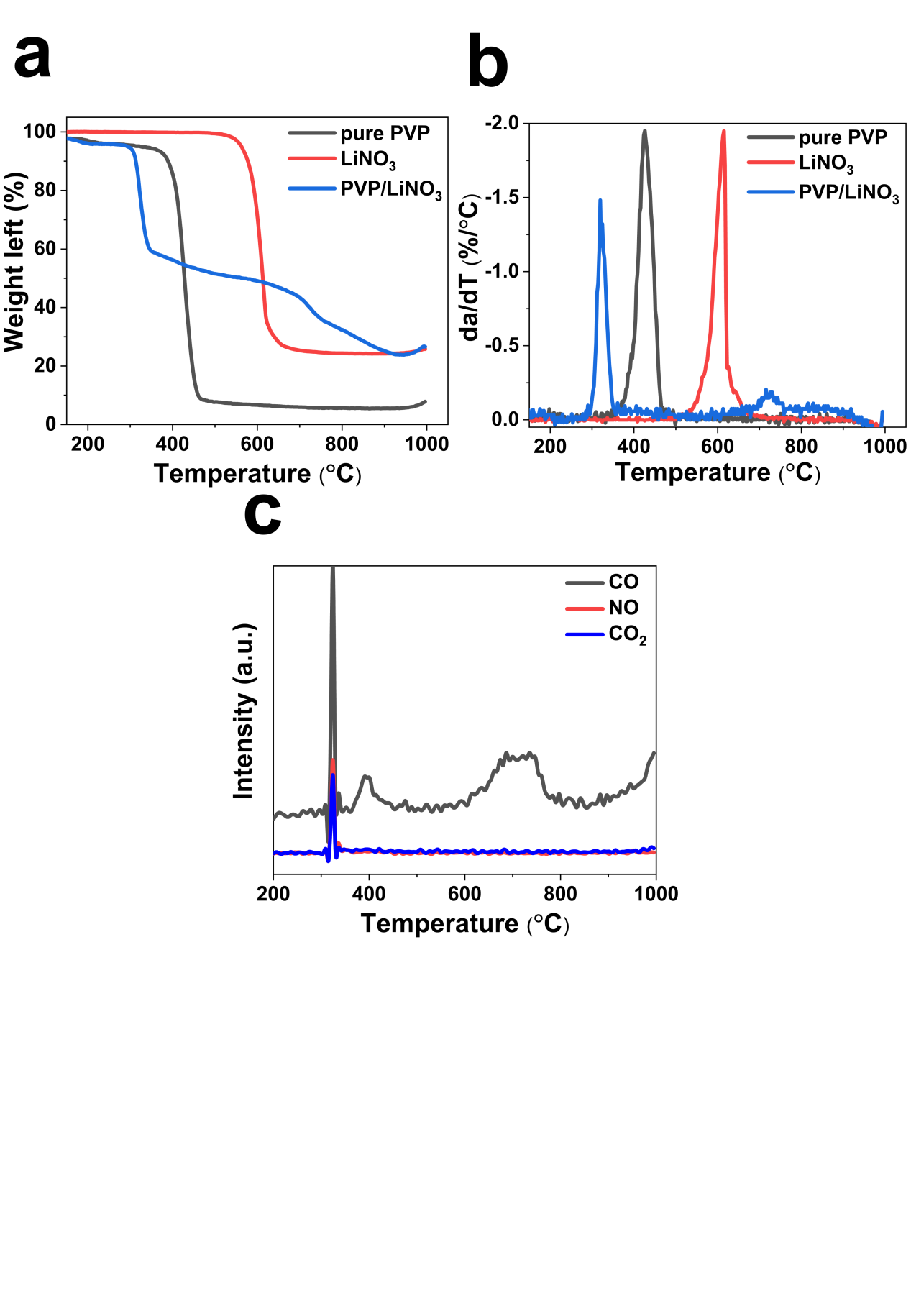


**Fig. S3.** (**a**) TGA and (**b**) DTG curves of PVP, LiNO_3_ and PVP/LiNO_3_. (**c**) TG-MS of the released gases from the pyrolysis of PVP/LiNO_3_.


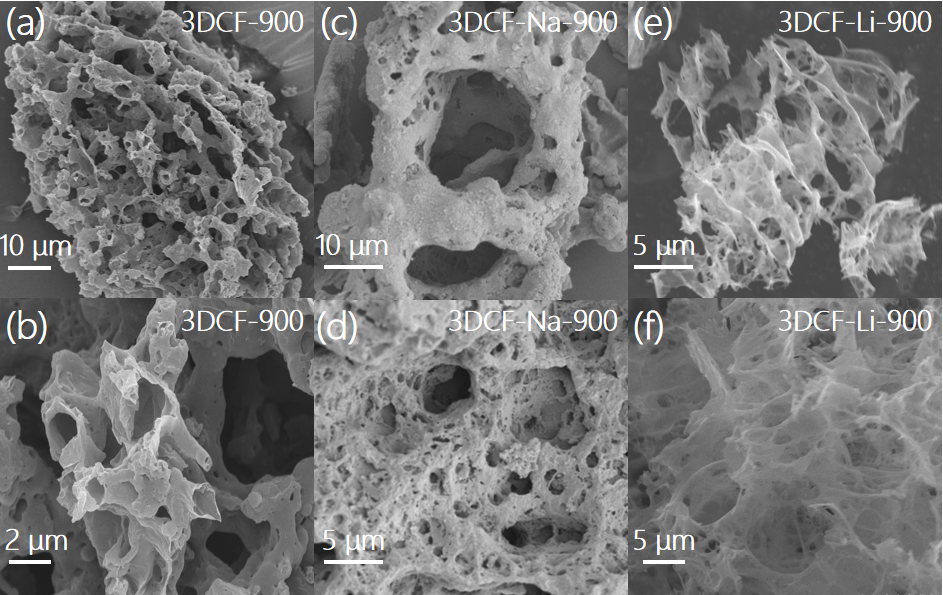


**Fig. S4.** SEM images of (**a**-**b**) 3DCF-900, (**c**-**d**) 3DCF-Na-900, (**e**-**f**) 3DCF-Li-900.


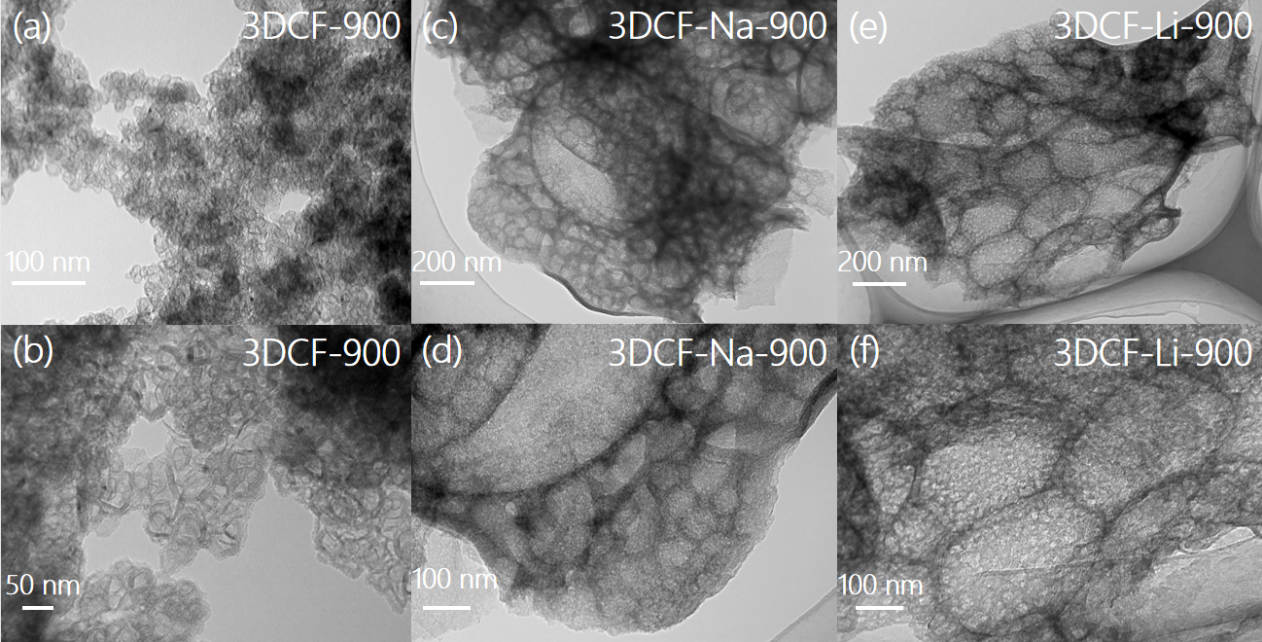


**Fig. S5.** TEM images of (**a**-**b**) 3DCF-900, (**c**-**d**) 3DCF-Na-900, (**e**-**f**) 3DCF-Li-900, indicating that the structures of 3DCF can be controlled by using different alkali metal nitrates (NaNO_3_, LiNO_3_).


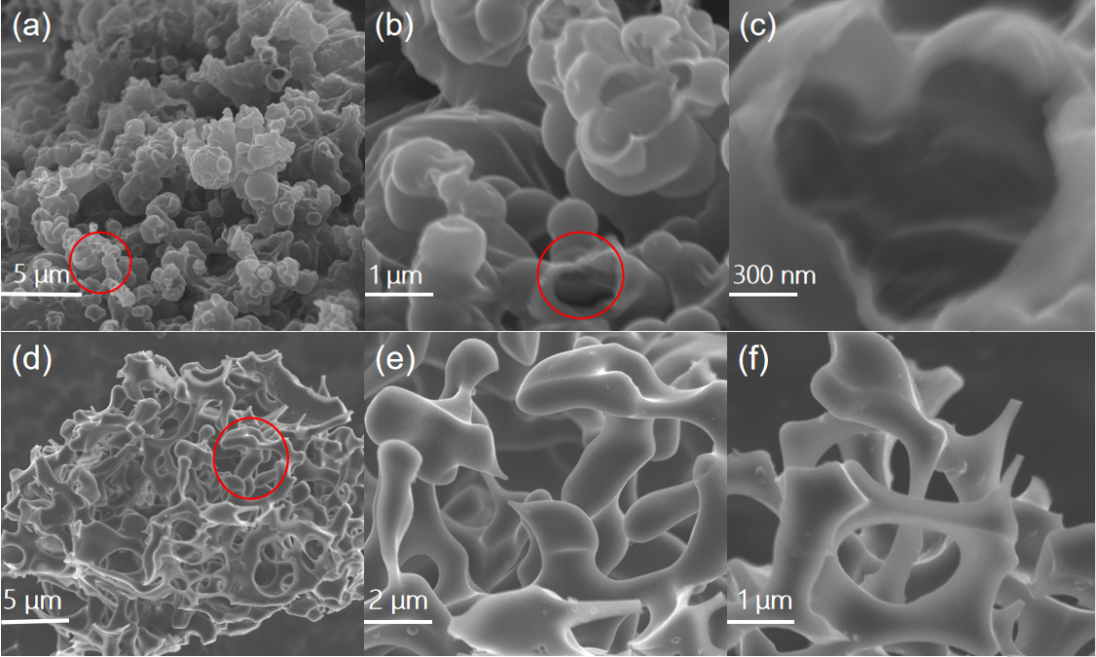


**Fig. S6.** SEM images of (**a-c**) 3D carbon frameworks with continuous nanocages by *in-situ* activation of KNO_3_; (**d-f**) 3DCFs by activation of KOH.


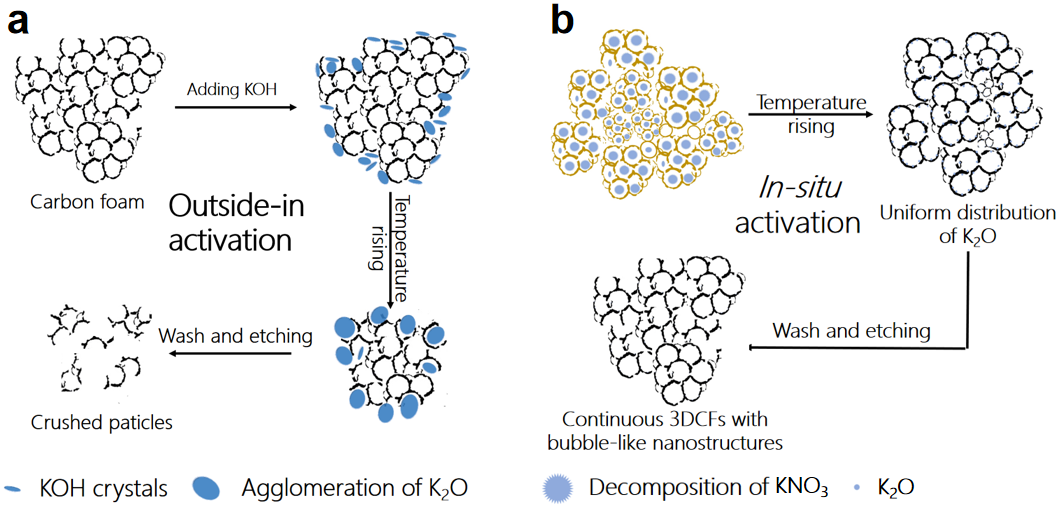


**Fig. S7.** The difference between mechanism of (**a**) traditional activation by KOH and (**b**) *in-situ* activation by uniform distribution of alkali metal oxides (AMOs).

The formation pores might undergo an expansion-etching mechanism on the basis of the following equations started above its decomposition temperature above 450 °C.

Expansion: 12 KNO_3_→6K_2_O+N_2_↑+10O_2_↑+10NO↑ (1)

Etching: 2K_2_O+C→4K+ CO_2_↑ (2)

CO_2_↑+C→2CO↑  (3)


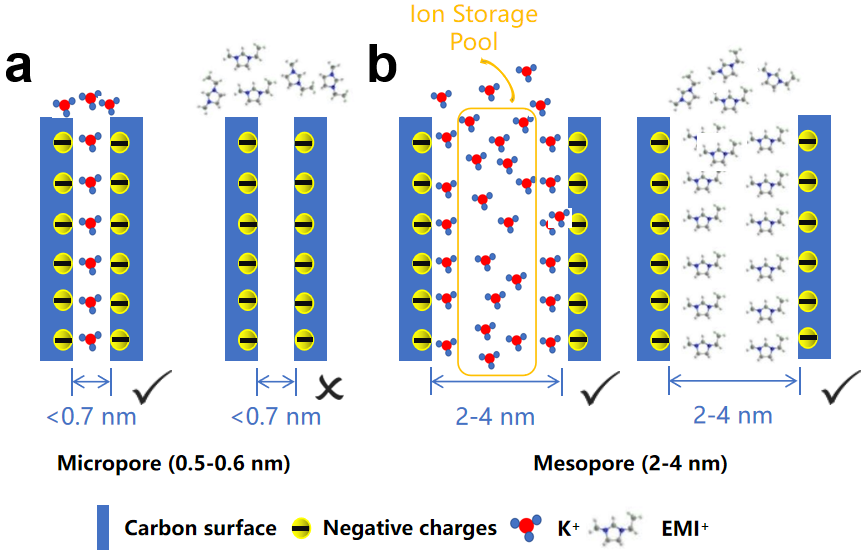


**Fig. S8.** The proposed ion diffusion models of EMI**^+^** in pores with different size. (a) < 0.7 nm; (b) 2-4 nm.

Ions in KOH electrolyte are small (0.3-0.45 nm for hydrated K and 0.6-0.65 nm for hydrated OH). Therefore, KOH based supercapacitors usually work well in terms of capacitance and its high-current retention with well selected microporous carbons without much need for mesopores. However, the mesopores of 3DCFs can provide accessible space for rapid ion buffer and energy storage even using EMI**^+^** ions (~0.7 nm) as electrolyte. When the pore size was smaller than 0.7 nm, the pore was inaccessible and useless to store energy in Fig. S8.


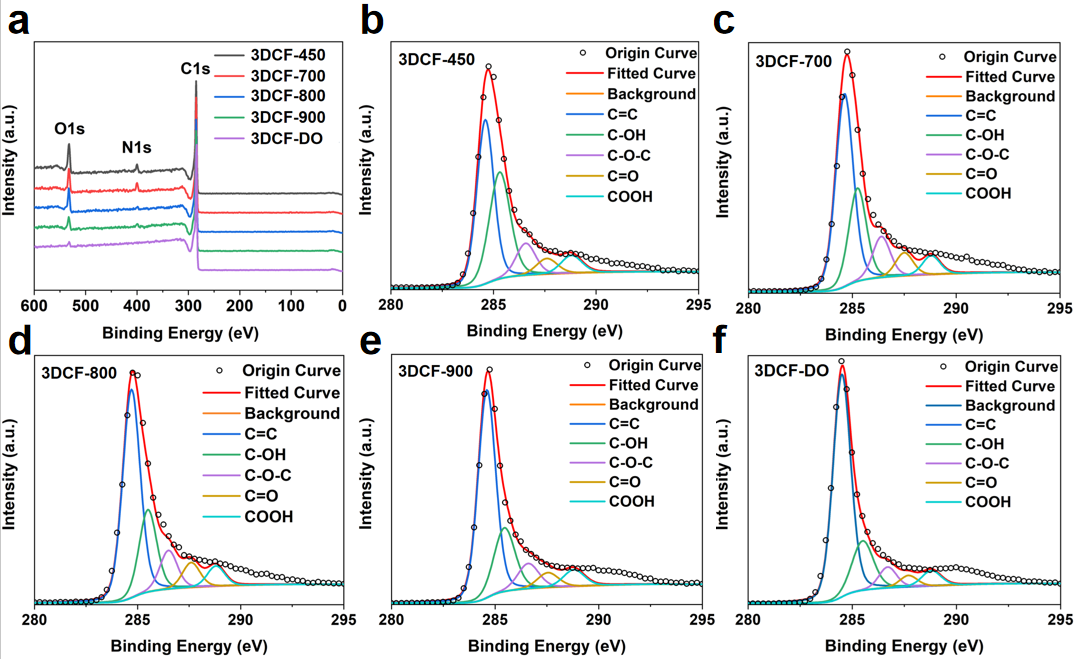


**Fig. S9.** X-ray photoelectron spectroscopy (XPS) spectra of the 3DCF materials. (**a**) Survey spectra and (**b-f**) C1s deconvoluted spectra of 3DCF-450, 3DCF-700, 3DCF-800, 3DCF-900 and 3DCF-DO, respectively.


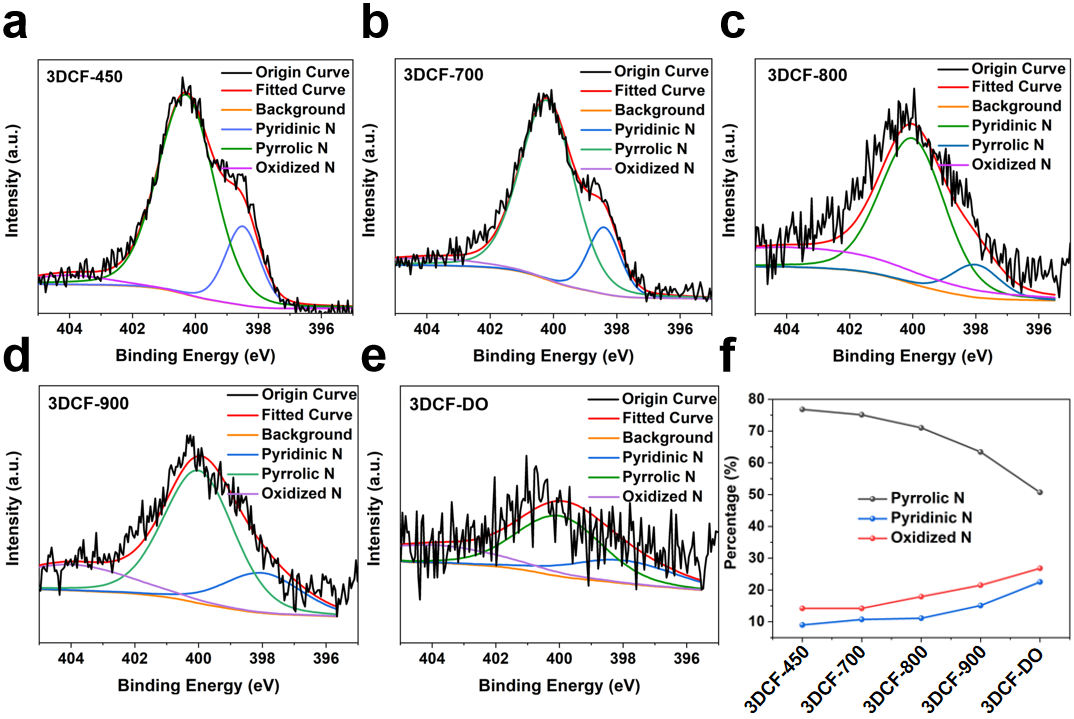


**Fig. S10.** (**a-e**) N1s deconvoluted spectra of 3DCF materials, (**f**) The change of various N species with annealing temperature.


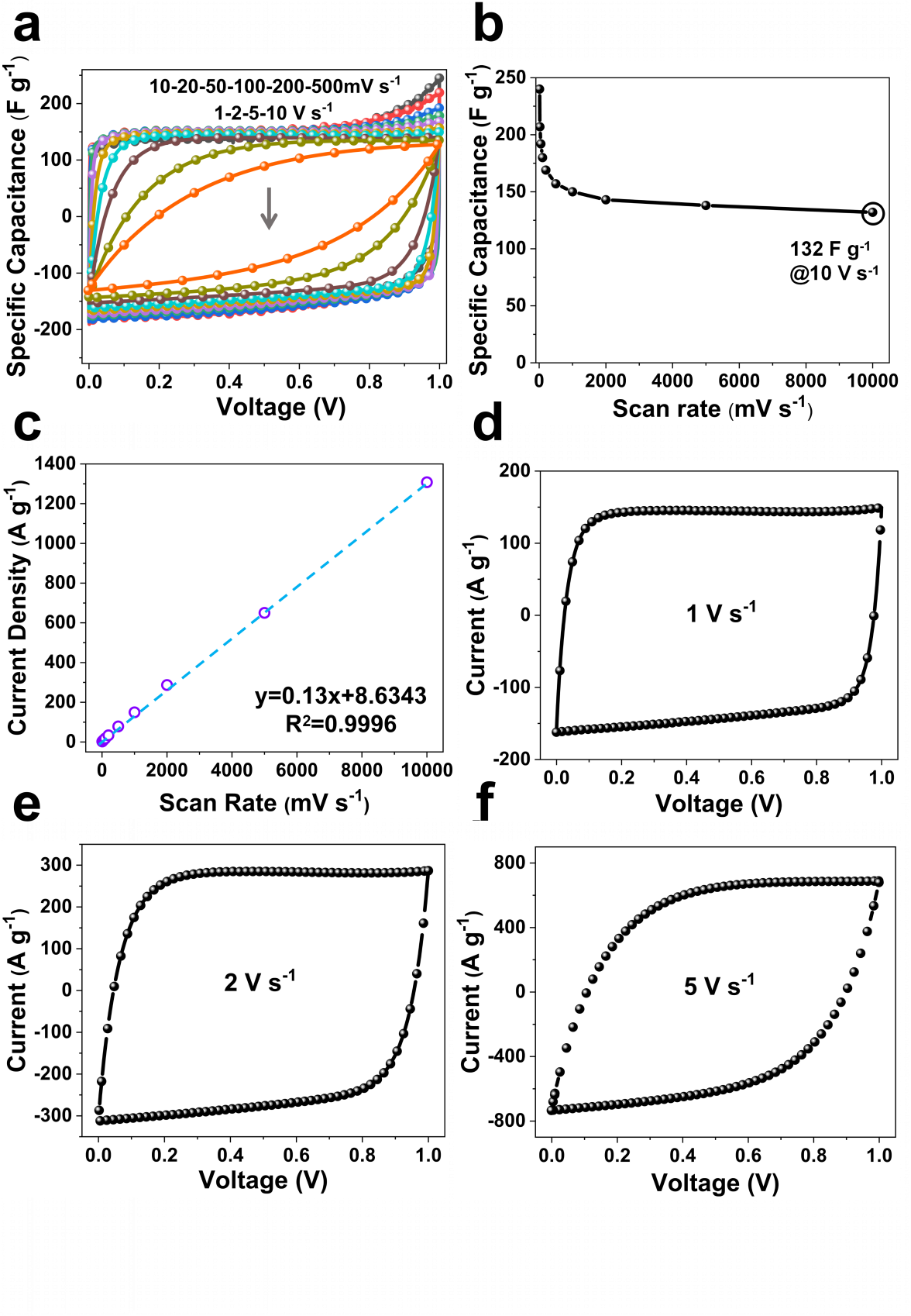


**Fig. S11.** Cyclic voltammetry performance of 3DCF-DO as electrode of symmetric two-electrode coin cell in 6 M KOH; (**a**) CV curves of various scan rates from 10 mV s^-1^ to 10 V s^-1^; (**b**) Rate performance from 10 mV s^-1^ to 10 V s^-1^; (**c**) Discharge current versus scan rate. A linear dependence is obtained at scan rates up to 10 V s^-1^ in the capacitive region. (**d-f**) CV curves of 3DCF-DO at different scan rates of 1 V s^-1^, 2 V s^-1^, 5 V s^-1^, respectively.


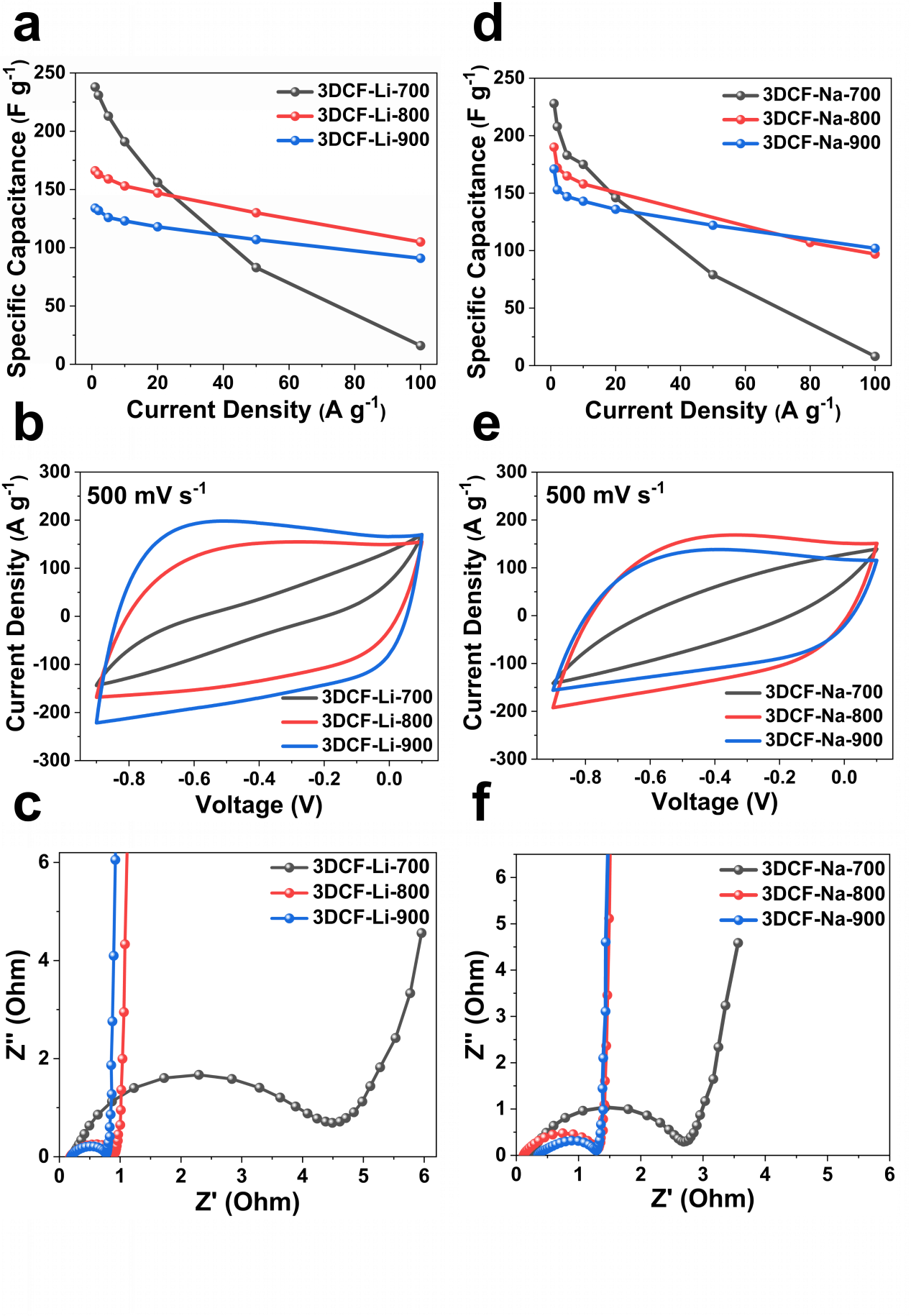


**Fig. S12.** Electrochemical performance characteristics of 3DCF-Li and 3DCF-Na materials measured in a two-electrode system in 6 M KOH; (**a**) Rate performance, (**b**) CV curves and (**c**) Nyquist plots of 3DCF-Li materials. (**d**) Rate performance, (**e**) CV curves and (**f**) Nyquist plots of 3DCF-Na materials.


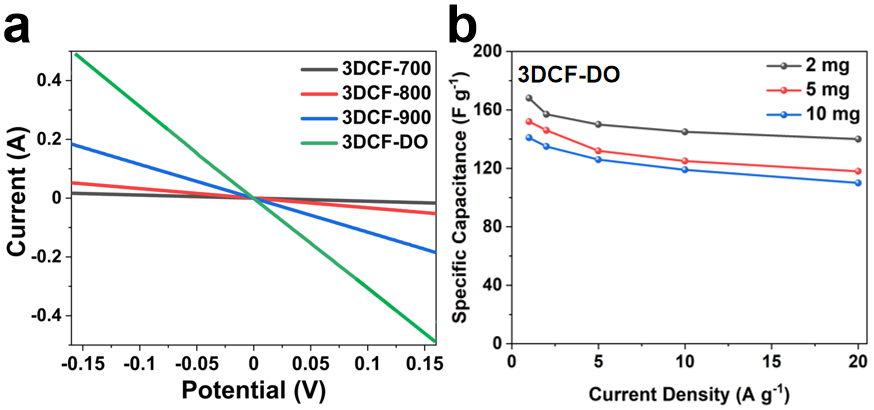


**Fig. S13.** The relationship between interconnected nanocages and corresponding materials properties, such as (a) LSV curves of 3DCF-700, 800, 900 and 3DCF-DO; (b) 3DCF-DO-based supercapacitors in 6 M KOH at various mass loadings of 2, 5 and 10 mg cm^-2^.


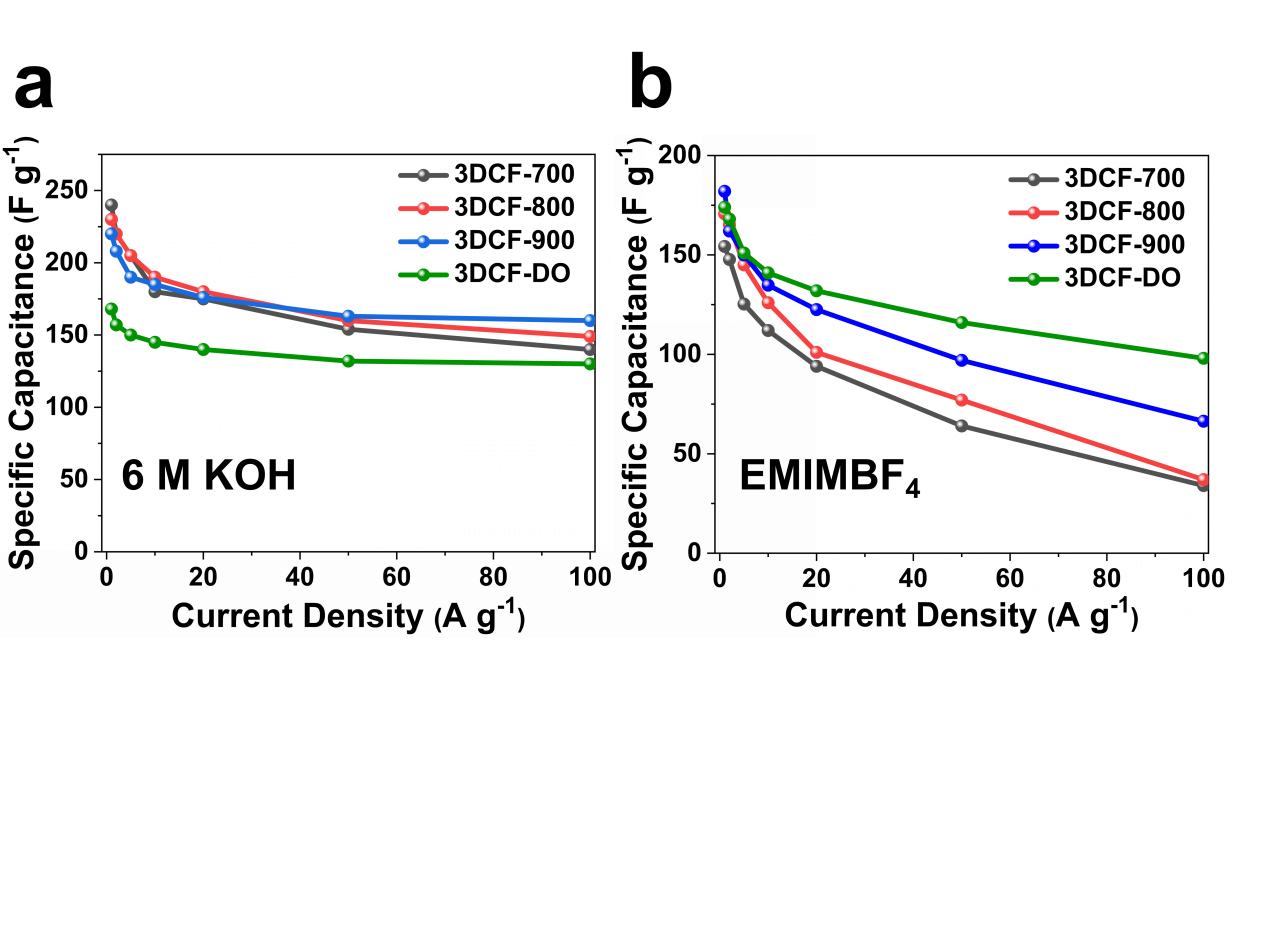


**Fig. S14.** Rate performance of 3DCF materials (3DCF-700, 3DCF-800, 3DCF-900 and 3DCF-DO) in (**a**) 6 M KOH and (**b**) EMIMBF_4_ electrolyte.


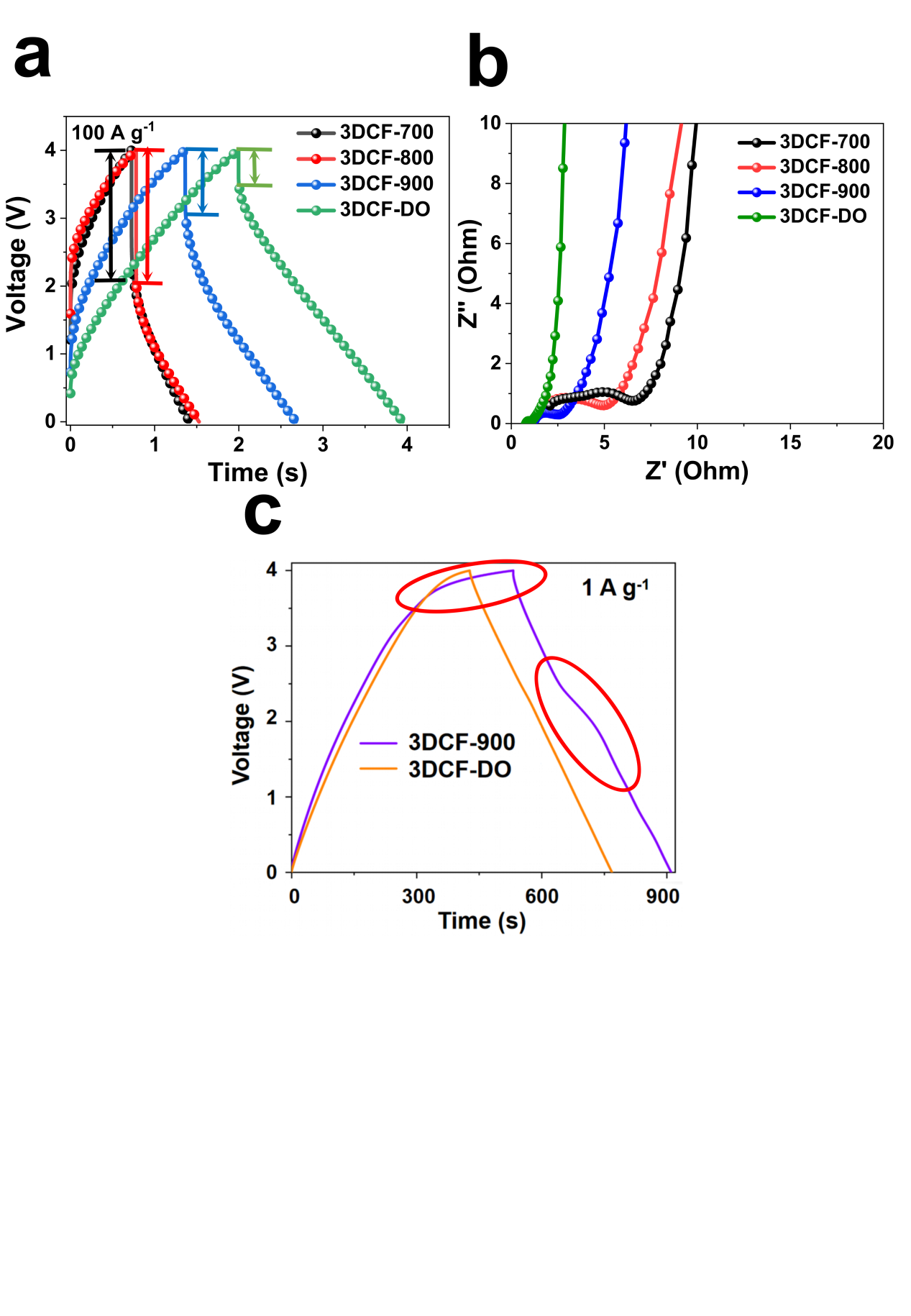


**Fig. S15.** Electrochemical performance of 3DCFs in a symmetric two-electrode coin cells in EMIMBF_4_ at 4 V; (**a**) IR drop, (**b**) Nyquist plots of 3DCF-700, 3DCF-800, 3DCF-900 and 3DCF-DO, (**c**) The electrochemical polarization in GCD curves of 3DCF-900 and 3DCF-DO at the current density of 1 A g^-1^. (Two obvious peaks for O decomposition and side reactions disappeared even at the small current density of 1 A g^-1^ after the deoxidization process.)

**
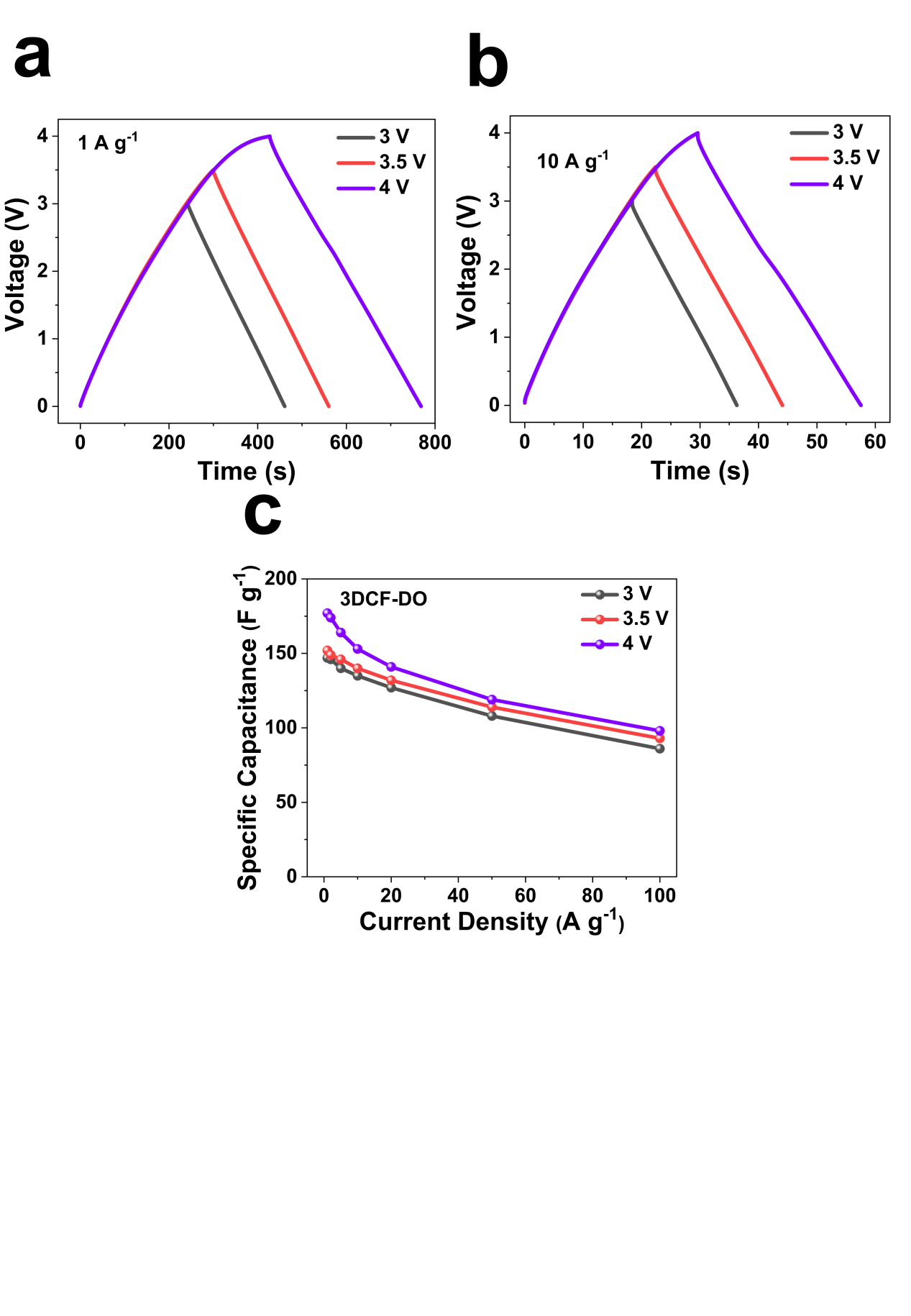
**

**Fig. S16.** 3DCF-DO-based supercapacitor at different potential windows of 3 V, 3.5 V, 4 V in EMIMBF_4_. (**a**) At the current density of 1 A g^-1^; (**b**) At the current density of 10 A g^-1^; (**c**) Rate performance at 3 V, 3.5 V and 4 V.

Table S1. Structural characteristics of 3DCF materials.

| Materials | SSA  (m^2^ g^-1^) | Pore volume (cm^3^ g^-1^) | V_meso_  (cm^3^ g^-1^)* | V_micro_  (cm^3^ g^-1^)* | V_meso_/V_micro_ | Average pore width (nm) |
| --- | --- | --- | --- | --- | --- | --- |
| 3DCF-450 | 1502 | 1.44 | 0.71 | 0.43 | 1.65 | 3.83 |
| 3DCF-700 | 2098 | 1.83 | 0.89 | 0.72 | 1.24 | 3.50 |
| 3DCF-800 | 2592 | 1.97 | 1.02 | 0.71 | 1.44 | 3.49 |
| 3DCF-900 | 2602 | 2.29 | 1.24 | 0.56 | 2.21 | 3.04 |
| 3DCF-DO | 2612 | 2.32 | 1.26 | 0.60 | 2.10 | 3.02 |

*: V_meso_: mesopore volume (2-50 nm); V_micro_: micropore volume (0-2 nm);

Table S2. Contents of C, N, O in 3DCF materials based on XPS.

| Materials (at%) | 3DCF-450 | 3DCF-700 | 3DCF-800 | 3DCF-900 | 3DCF-DO |
| --- | --- | --- | --- | --- | --- |
| C | 83.07 | 86.35 | 90.42 | 93.09 | 97.45 |
| N | 5.6 | 5.02 | 1.68 | 1.11 | 0.86 |
| O | 11.33 | 8.63 | 7.90 | 5.80 | 1.69 |

Table S3. C1s analysis of 3DCF materials.

| Materials (at%) | C=C  (284.5 eV) | C-OH  (285.4 eV) | C-O-C  (286.8 eV) | C=O  (287.3 eV) | COOH  (288.8 eV) |
| --- | --- | --- | --- | --- | --- |
| 3DCF-450 | 57.57 | 23.77 | 8.60 | 4.88 | 5.18 |
| 3DCF-700 | 58.70 | 19.17 | 9.04 | 6.79 | 6.30 |
| 3DCF-800 | 63.13 | 16.72 | 8.92 | 5.92 | 5.31 |
| 3DCF-900 | 66.38 | 16.43 | 7.05 | 4.86 | 5.28 |
| 3DCF-DO | 70.11 | 15.37 | 6.54 | 4.53 | 3.45 |

Table S4. Various resistances of the 3DCF materials in ILs electrolyte.

| Materials | ESR (Ω) | Rs (Ω) | Rct (Ω) |
| --- | --- | --- | --- |
| 3DCF-700 | 10.75 | 2.41 | 2.74 |
| 3DCF-800 | 5.51 | 1.69 | 2.22 |
| 3DCF-900 | 3.94 | 1.37 | 0.90 |
| 3DCF-DO | 2.43 | 0.80 | 0.18 |

Table S5. The corresponding relaxation time constant (τ, s) of the 3DCF materials.

| Electrolyte | 3DCF-700 | 3DCF-800 | 3DCF-900 | 3DCF-DO |
| --- | --- | --- | --- | --- |
| 6 M KOH | 0.94 s | 0.43 s | 0.29 s | 0.19 s |
| EMIMBF_4_ | 3.73 s | 3.54 s | 2.50 s | 0.89 s |

Table S6. Comparison of 3DCF materials with other advanced carbon electrodes for aqueous SCs.

| Materials | S_BET_ (m^2^ g^-1^) | C (F g^-1^) | Potential (V) | Current Density (A g^-1^) | Electrolyte | Ref. |
| --- | --- | --- | --- | --- | --- | --- |
| 3DCF-900 | 2602 | 220 (160) | 1 | 1 (100) | 6 M KOH | This work |
| 3DCF-DO | 2612 | 168 (130) | 1 | 1 (100) | 6 M KOH | This work |
| ZNG | 2020 | 336 | 1 | 0.5 | 1 M H_2_SO_4_ | S1 |
| CoDCs | 1730 | 270 (120) | 1 | 1 (100) | 6 M KOH | S2 |
| OMC-1 | 650 | 208 (106) | 1 | 0.2 (100) | 6 M KOH | S3 |
| EM-CCG | 962 | 203 (145) | 1 | 0.1 (100) | 1 M H_2_SO_4_ | S4 |
| KNOSC | 2685 | 403 (309) | 1 | 1 (100) | 6 M KOH | S5 |
| GO-160-8D | 305 | 436 (261) | 1 | 0.5 (50) | 6 M KOH | S6 |

Table S7. Comparison of 3DCF materials with other advanced carbon electrodes for ILs-based SCs.

| Materials | S_BET_  (m^2^ g^-1^) | Energy Density  (Wh kg^-1^) | Power Density  (kW kg^-1^) | Potential (V) | Electrolyte | Ref. |
| --- | --- | --- | --- | --- | --- | --- |
| 3DCF-900 | 2602 | 100 (1.46) | 1 (150) | 4 | EMIMBF_4_ | This work |
| 3DCF-DO | 2612 | 97 (34) | 1 (150) | 4 | EMIMBF_4_ | This work |
| 3DCF-DO(40 ºC) | 2612 | 108.3 (58.3) | 1 (100) | 4 | EMIMBF_4_ | This work |
| 3DCF-DO(60 ºC) | 2612 | 127.8 (62.8) | 1 (100) | 4 | EMIMBF_4_ | This work |
| BNP-HGH | 980 | 38.5 | 83 | 3.5 | EMIMBF_4_ | S7 |
| AHPC | 2650 | 67 | 18 | 3.6 | EMIMBF_4_ | S8 |
| PC-1000 | 2096 | 65 | 81.5 | 3.5 | EMIMBF_4_ | S9 |
| GNFs | 1280 | 55.4 | 20 | 4 | EMIMBF_4_ | S10 |
| GMS sheet | 1500 | 48 | 20 | 4.4 | TEABF_4_ /PC | S11 |

Table S8. Comparison of mass loading performance for various carbon-based SCs in aqueous electrolytes.

| Materials | Mass loading  (mg cm^-2^) | C_g_  (F g^-1^) |
| --- | --- | --- |
| 3DCF-DO  (this work) | 2  5  10 | 168 (1 A g^-1^)  155 (1 A g^-1^)  142 (1 A g^-1^) |
| Activated carbon [S12] | 5-10 | 80-120 |
| EM-CCG film [S13] | 1  10 | 167 (1 A g^-1^)  126 (1 A g^-1^) |
| Compressed MEGO [S14] | 4.3 | 147 (1.2 A g^-1^) |
| Graphene framework [S15] | 1  10 | 289 (1 A g^-1^)  246 (1 A g^-1^) |
| CNT arrays [S16] | 5 | 160 (1 A g^-1^) |
| Actived grephene [S17] | 4 | 231 (1 A g^-1^) |
| CCNC3 [S18] | 3.5  10.6 | 177 (1 A g^-1^)  156 (1 A g^-1^) |
| Laser scribed graphene [S19] | 0.036 | 276 (5 A g^-1^) |

**References and notes**

[S1] X. Jiang, R. Li, M. Hu, Z. Hu, D. Golberg, Y. Bando, X. Wang. Zinc-Tiered Synthesis of 3D Graphene for Monolithic Electrodes. Adv. Mater. **31**, 11901186 (2019). <https://doi.org/>[10.1002/adma.201901186](https://www.x-mol.com/paperRedirect/5672678).

[S2] S. Zhang, J. Zhu, Y. Qing, et al. Ultramicroporous Carbons Puzzled by Graphene Quantum Dots: Integrated High Gravimetric, Volumetric, and Areal Capacitances for Supercapacitors. Adv. Funct. Mate*r.* **28**, 1805898 (2018). [https://doi.org/10.1002/adfm. 201805898](https://doi.org/10.1002/adfm.201805898).

[S3] X. Xi, D. Wu, L. Han, Y. Yu, Y. Su, W. Tang, R. Liu. Highly Uniform Carbon Sheets with Orientation-Adjustable Ordered Mesopores. ACS Nano **12**, 5436-5444 (2018). https: //doi.org/[10.1021/acsnano.8b00576](https://www.x-mol.com/paperRedirect/661233).

[S4] X.Yang, C. Cheng, Y. Wang, L. Qiu and D. Li. Liquid-mediated dense integration of graphene materials for compact capacitive energy storage. Science **341**, 534-537 (2013). https://doi.org/[10.1126/science.1239089](https://www.x-mol.com/paperRedirect/1771345).

[S5] H. Peng, B. Yao, X. Wei, T. Liu, T. Kou, P. Xiao, Y. Zhang, Y. Li. Pore and Heteroatom Engineered Carbon Foams for Supercapacitors. Adv. Energy Mater. **9**, 1803665 (2019). <https://doi.org/>[10.1002/aenm.201803665](https://www.x-mol.com/paperRedirect/5618487).

[S6] Z. Liu, L. Jiang, L. Sheng, Q. Zhou, T. Wei, B. Zhang, Z. Fan. Oxygen Clusters Distributed in Graphene with "Paddy Land" Structure: Ultrahigh Capacitance and Rate Performance for Supercapacitors. Adv. Funct. Mater. **28**, 1705258 (2017). <https://doi.org/> [10.1002/adfm.201705258](https://www.x-mol.com/paperRedirect/466566).

[S7] Z. Pan, H. Zhi, Y. Qiu, J. Yang, L. Xing, Q. Zhang, X. Ding, X. Wang, G. Xu, H. Yuan, M. Chen, W. Li, Y. Yao, N. Motta, M. Liu, Y. Zhang. Achieving commercial- level mass loading in ternary-doped holey graphene hydrogel electrodes for ultrahigh energy density supercapacitors. Nano Energy **46**, 266 (2018). <https://doi.org/>[10.1016/j.nanoen. 2018.02.007](https://www.x-mol.com/paperRedirect/547038).

[S8] G. Zhao, C. Chen, D. Yu, L. Sun, C. Yang, H. Zhang, Y. Sun, F. Besenbacher, M. Yu. One-Step Production of O-N-S Co-Doped Three-Dimensional Hierarchical Porous Carbons for High-Performance Supercapacitors. Nano Energy **47**, 547 (2018). [https:// doi.org/](https://doi.org/)[10.1016/j.nanoen.2018.03.016](https://www.x-mol.com/paperRedirect/591543).

[S9] J. Niu, R. Shao, J. Liang, M. Dou, Z. Li, Y. Huang and F. Wang. Biomass- derived mesopore-dominant porous carbons with large specific surface area and high defect density as high performance electrode materials for Li-ion batteries and supercapacitors. Nano Energy **36**, 322-330 (2017).<https://doi.org/>[10.1016/j.nanoen. 2017.04.042](https://www.x-mol.com/paperRedirect/250150)

[S10] C. Cui, W. Qian, Y. Yu, C. Kong, B. Yu, F. Wei. Highly Electroconductive Mesoporous Graphene Nanofibers and Their Capacitance Performance at 4 V. J. Am. Chem. Soc. **136**, 2256-2259 (2014). <https://doi.org/> [10.1021/ja412219r](https://www.x-mol.com/paperRedirect/1810320).

[S11] K. Nomura, H. Nishihara, N. Kobayashi, T. Kyotani. 4.4 V supercapacitors based on super-stable mesoporous carbon sheet made of edge-free graphene walls. Energy Environ. Sci. **12**, 1542 (2019). <https://doi.org/>[10.1039/c8ee03184c](https://www.x-mol.com/paperRedirect/963235).

[S12] A. Burke. R&D considerations for the performance and application of electrochemical capacitors. Electrochim. Acta **53**, 1083 (2007). [https://doi.org/10.1016/j. electacta.](https://doi.org/10.1016/j.electacta.)2007.01.011.

[S13] X. Yang, C. Cheng, Y. Wang, L. Qiu, D. Li. [Liquid-Mediated Dense Integration of Graphene Materials for Compact Capacitive Energy Storage](http://med.wanfangdata.com.cn/Paper/Detail/PeriodicalPaper_PM23908233). Science **341**, 534 (2013). https://doi.org/10.1126/science.1239089.

### [S14] S. Murali, N. Quarles, L. L. Zhang, J. R. Potts, Z. Tan, Y. Lu, Y. Zhu, R. S. Ruoff. [Hydrothermal synthesis of macroscopic nitrogen-doped graphene hydrogels for ultrafast supercapacitor](http://www.sciencedirect.com/science/article/pii/S2211285512001772). Nano Energy 2, 764 (2013). <https://doi.org/10.1016/j.nanoen.>2012.09. 003.

[S15] X. Wang, Y. Zhang, C. Zhi, X. Wang, D. Tang, Y. Xu, Q. Weng, X. Jiang, M. Mitome, D. Golberg, Y. Bando. [Three-dimensional strutted graphene grown by substrate-free sugar blowing for high-power-density supercapacitors](https://xueshu.baidu.com/usercenter/paper/show?paperid=dabd00f8fcbef349673fe67fe4012c24&site=xueshu_se&sc_from=dlut). Nat. Commun. **4**, 2905-2913 (2013). <https://doi.org/10.1038/ncomms3905>.

[S16] A. Izadi-Najafabadi, S. Yasuda, K. Kobashi, T. Yamada, D. N. Futaba, H. Hatori, M. Yumura, S. Iijima, K. Hata. [Extracting the Full Potential of Single-Walled Carbon Nanotubes as Durable Supercapacitor Electrodes Operable at 4 V with High Power and Energy Density](http://med.wanfangdata.com.cn/Paper/Detail/PeriodicalPaper_PM20564700). Adv. Mater. **22**, E235 (2010). https://doi.org/10.1002/adma.200904349.

### [S17] L. Zhang, F. Zhang, X. Yang, G. Long, Y. Wu, T. Zhang, K. Leng, Y. Huang, Y. Ma, A. Yu, Y. Chen. [Porous 3D graphene-based bulk materials with exceptional high surface area and excellent conductivity for supercapacitors](http://www.ncbi.nlm.nih.gov/pubmed/23474952). Sci. Rep. 3, 1408 (2013). https://doi.org/10. 1038/srep01408.

### [S18] Bu Y, Sun T, Cai Y. [Compressing Carbon Nanocages by Capillarity for Optimizing Porous Structures toward Ultrahigh-Volumetric-Performance Supercapacitor.](http://www.researchgate.net/publication/316207680_Compressing_Carbon_Nanocages_by_Capillarity_for_Optimizing_Porous_Structures_toward_Ultrahigh-Volumetric-Performance_Supercapacitors) Adv. Mater. 29, 1700470.1-1700470.7. (2017). https://doi.org/10.1002/adma.201700470.

[S19] M. F. El-Kady, V. Strong, S. Dubin, R. B. Kaner. Laser scribing of high- performance and flexible graphene-based electrochemical capacitors. Science **335**, 1326 (2012). https://doi.org/10.1126/science.1216744
